# Diversity of Marine-Derived Fungal Cultures Exposed by DNA Barcodes: The Algorithm Matters

**DOI:** 10.1371/journal.pone.0136130

**Published:** 2015-08-26

**Authors:** Nikos Andreakis, Lone Høj, Philip Kearns, Michael R. Hall, Gavin Ericson, Rose E. Cobb, Benjamin R. Gordon, Elizabeth Evans-Illidge

**Affiliations:** Australian Institute of Marine Science, PMB 3, Townsville, Queensland, 4810, Australia; U.S. Geological Survey, UNITED STATES

## Abstract

Marine fungi are an understudied group of eukaryotic microorganisms characterized by unresolved genealogies and unstable classification. Whereas DNA barcoding via the nuclear ribosomal internal transcribed spacer (ITS) provides a robust and rapid tool for fungal species delineation, accurate classification of fungi is often arduous given the large number of partial or unknown barcodes and misidentified isolates deposited in public databases. This situation is perpetuated by a paucity of cultivable fungal strains available for phylogenetic research linked to these data sets. We analyze ITS barcodes produced from a subsample (290) of 1781 cultured isolates of marine-derived fungi in the Bioresources Library located at the Australian Institute of Marine Science (AIMS). Our analysis revealed high levels of under-explored fungal diversity. The majority of isolates were ascomycetes including representatives of the subclasses Eurotiomycetidae, Hypocreomycetidae, Sordariomycetidae, Pleosporomycetidae, Dothideomycetidae, Xylariomycetidae and Saccharomycetidae. The phylum Basidiomycota was represented by isolates affiliated with the genera *Tritirachium* and *Tilletiopsis*. BLAST searches revealed 26 unknown OTUs and 50 isolates corresponding to previously uncultured, unidentified fungal clones. This study makes a significant addition to the availability of barcoded, culturable marine-derived fungi for detailed future genomic and physiological studies. We also demonstrate the influence of commonly used alignment algorithms and genetic distance measures on the accuracy and comparability of estimating Operational Taxonomic Units (OTUs) by the automatic barcode gap finder (ABGD) method. Large scale biodiversity screening programs that combine datasets using algorithmic OTU delineation pipelines need to ensure compatible algorithms have been used because the algorithm matters.

## Introduction

Marine microorganisms are biodiverse and globally important, accounting for 90% of ocean biomass and 98% of ocean respiration [1]. Marine fungi are part of the eukaryotic microbial biodiversity and as an ecological group, defined based on their habitat. They can be sub-divided into those that are fully adapted and require a marine environment to complete their life cycle (residents) and those that have been washed or blown from a freshwater or terrestrial environment and are able to survive by tolerating marine conditions (transients) [2]. Whereas obligate marine fungi can grow and sporulate exclusively in marine or estuarine environments, transients are facultative marine species which may also grow in freshwater and terrestrial habitats [3].

Marine fungi demonstrate a variety of habits and life cycle strategies, and include free-living, parasitic and widespread symbiotic forms. Although the ecological role of marine fungi is poorly understood, purported roles include degradation of biota, provision of chemical protection, pathogenicity, symbiosis and contribution to diverse holobiont communities [4–6]. Advancement in marine mycology is confounded by under-representation of fungal strains in culture collections, and the poor recovery of fungal SSU rRNA gene sequences in clone libraries from marine environmental samples [4].

While less studied than prokaryotic microorganisms, marine-derived fungi are emerging as a productive source of new natural products with fermentation, bioremediation and therapeutic potential [7, 8]. Over 60% of the 456 new marine microbial natural products reported in 2012, were produced by fungi [7]. Fungi also account for the highest number of novel compounds reported from sponge-associated microorganisms in the last decades [7, 9].

The Australian Institute of Marine Science (AIMS) Bioresources Library hosts a culture collection of over 7,000 un-sequenced microorganisms, including 1,781 marine-derived fungi isolated from various sources including invertebrates, sediment and seawater. In an attempt to identify bioactivity trends amongst higher macro-organism phyla represented in the AIMS Bioresources Library, a recent study analysed bioassay data with respect to taxonomy, phylogeny and bioregional origins [10]. That study incorporated bioactivity results from 18,000 individual bioassays of samples collected from over 1,200 sites across 10 Australian marine bioregions and 13 macro-organism phyla [10]. The study concluded that bioactivity was primarily explained by high level phylogenetic grouping rather than habitat diversity or specific ecological profiles. A similar analysis targeting microbial phyla has not been possible to date, due to incomplete taxonomic characterization of the microbial isolates.

Despite their putative ecological significance and growing importance to biodiscovery, fungal phylogeny and systematics remain largely underexplored [11, 12]. Classification of fungi traditionally relied on sexual characters or morphological and physiological criteria, many of which are unstable in culture conditions or do not reflect any genealogical relationships [13]. There is no unique accepted system of classification at the higher taxonomic level despite a large scale collaborative effort to do so [14]. Although presently classified into seven phyla, the adoption of an unambiguous species concept still remains disputable in fungal taxonomy. However, it has been argued that molecular identification of operational taxonomical units (OTUs) via DNA barcoding may be sufficient to provide both a link between fungal systematics and phylogenetics, and a powerful tool in large scale screening projects for the identification of new candidate lineages for biodiscovery [15].

DNA barcoding techniques have provided standardized, reliable and cost-effective methods for marine and terrestrial species identification [16–18]. The internal transcribed spacer (ITS) region of nuclear DNA (nrDNA) has been recently accepted by a multinational multilaboratory consortium as a suitable marker for barcoding fungi [19], although some primer combinations may apparently introduce taxonomic bias in some fungal groups [20]. Nevertheless, the ITS region has been successfully used for fungal species identification and phylogenetic inference, in addition to barcoding environmental DNA samples (eDNA), via high throughput next generation sequencing (NGS) technology [21–23]. This approach has immensely accelerated the discovery of new lineages and has consequently revolutionized our perception of spatial and temporal levels of diversity in fungi [21].

Biological sequence alignment followed by estimation of evolutionary distance, are the first two crucial steps in molecular systematics, phylogenetic inference and comparative genomics [24–27]. Barcoding analysis typically begins with a sequence alignment step followed by the application of a clustering algorithm using a genetic distance threshold determined *a priori*, for the calculation of genetic similarity between pairs of sequences and the establishment of barcode clusters referred to as species or OTUs. [16, 18]. There are several alternative ways of computing sequence alignments and estimating evolutionary distance, each developed to fit a variety of data and analytical purposes [28, 29]. While realistic evolutionary distances can be calculated *a priori* by specifying appropriate substitution models that best fit the data [30], the accuracy of common alignment algorithms and their influence on both the final tree topology and genetic divergence estimates for downstream OTU delineation and biodiversity conclusions remains controversial [27, 31].

In this paper the ITS marker is used to evaluate fungal diversity in a subset of 290 cryopreserved fungal isolates obtained from multiple marine sources. This study aims to characterize each isolate at the lowest possible taxonomic unit, to identify and document unknown fungal lineages and assess fungal diversity with respect to taxonomy, geography and source of the isolates analyzed. By using four different commonly used alignment algorithms and evolutionary distance estimators for species identification, this paper also aims to evaluate the respective influences, biases and limitations of these algorithms in automatic quantitative fungal OTU delineation from ITS barcodes.

## Materials & Methods

### Geographical sampling, culture isolation & growth

**Fig 1:**
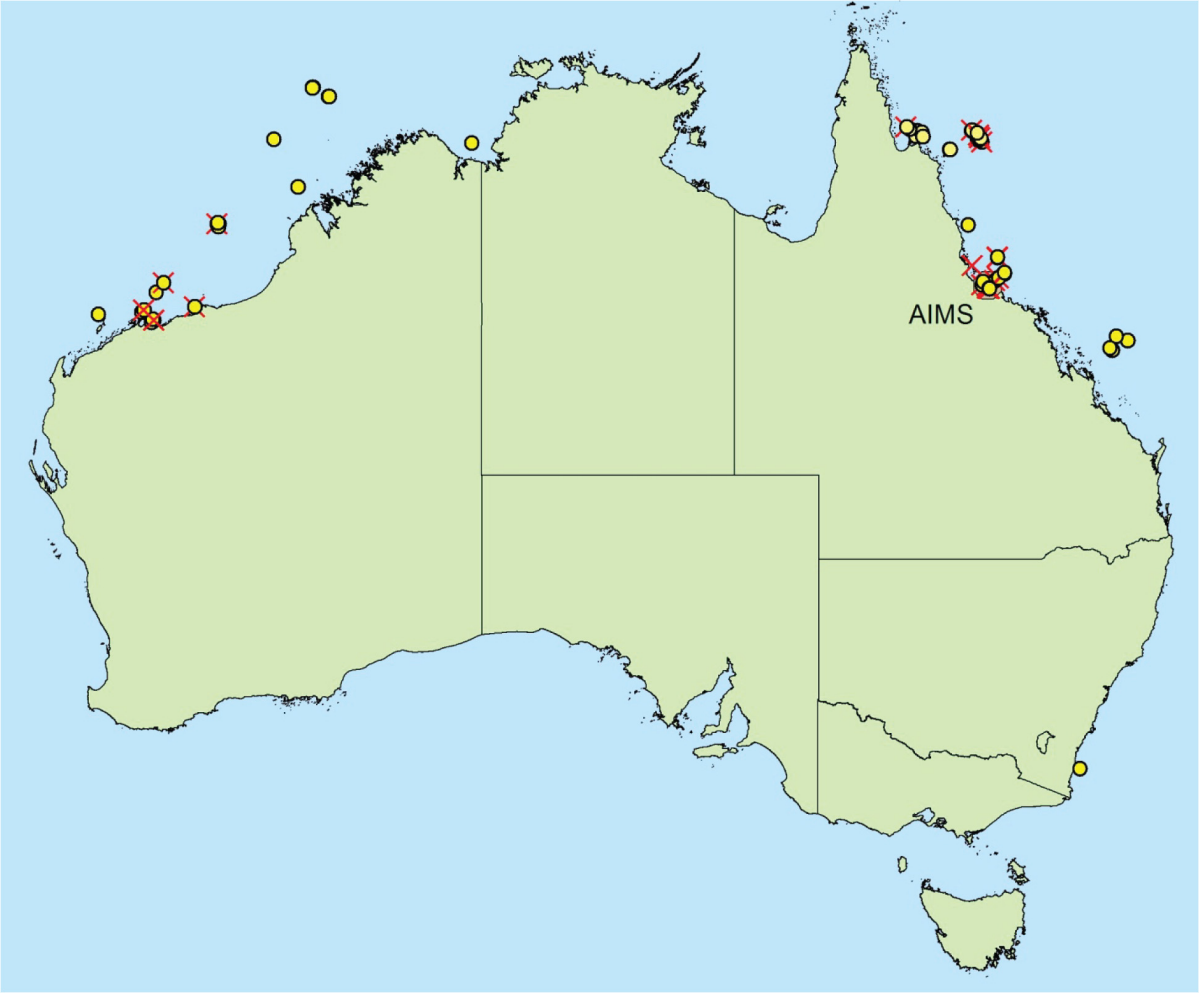
Specimen collection sites. Red crosses denote sites where unknown OTUs have been recovered. This figure was generated by the authors using ArcMap 10.1 with the Geodata Coast 100k base layer [74] available under creative commons 4.0 license.

All examined isolates were from the AIMS Bioresources library located at Cape Ferguson, Queensland, Australia. Source samples were legally collected between 1994 and 2008 under Great Barrier Reef Marine Park Authority (GBRMPA) permits (G94/587, G05/11866.1) from collection sites shown in Fig 1. Samples from sites outside the Great Barrier Reef Marine Park did not require permits at the time of collection. Depending on each isolate’s source jurisdiction (S1 Table), their future use is subject to either the benefit sharing agreement between the Australian Institute of Marine Science (AIMS) and the State of Queensland, or the deed of benefit-sharing between AIMS and the Commonwealth of Australia. S2 Table details each strain’s accession number, source material, collection location, distance to nearest landmass or island, and salinity condition of inshore sites. Sample collection, processing, isolation and culture methods were described in detail previously [32]. The majority of samples were plated on seawater-based media including filter paper moistened with sterile seawater, malt extract agar, yeast peptone agar, potato carrot agar [32] and diluted brain heart infusion agar (Brain Heart Infusion (Difco, BD) 3.7 g, bacto agar 10 g, seawater to 1 L). Only seven of the isolates grown with brain heart infusion agar were prepared with mQ water instead of seawater (S1 Table). Each agar medium was amended with antibiotics (Streptomycin 10 mg/L or Penicillin G 300 mg/L and Streptomycin 500 mg/L).

### DNA extraction, PCR amplification & sequencing

Total genomic DNA was extracted from the 290 fungal isolates using the Power Plant DNA Isolation Kit (MoBio Laboratories Inc., Carlsbad, CA) following the manufacturer’s recommendations. Quality and quantity of the extracted DNA was assessed in 1% agarose gel against known standards. The nuclear first internal transcribed spacer (ITS-1), the 5.8S gene and the second internal transcribed spacer were PCR amplified using primers ITS1 and ITS4 [33]. PCR reactions contained approximately 5 ng of DNA template, 10 μl 5x MyTaq Reaction Buffer, 0.15 μl of each primer (100 pmol μl^-1^), 0.4 μl of bovine serum albumin (BSA; 10 mg ml^-1^), 3 μl MgCl_2_ (50 mM), and 0.2 μl of MyTaq polymerase (Bioline, London, UK). The thermal cycling included: 1 cycle at 95°C for 5 min; 30 cycles at 94°C for 50 sec, 55°C for 50 sec, 72°C for 1.5 min; and a final elongation at 72°C for 10 min. PCR products were sent to Macrogen Inc. (Seoul, Korea) for purification and sequencing in both directions with the same primers used for the PCR reactions. Sequence accession numbers (KP890357—KP890646) are reported in S2 Table).

### Sequence alignments, BLAST searches, data exploration & genetic distance computations

Electropherograms were assembled in Sequencher 4.9 (Gene Codes) and a preliminary alignment was computed and trimmed in Bioedit v7.0.9 [34] using the ClustalW algorithm. BLAST searches were performed against publically available nucleotide databases (at: http://blast.ncbi.nlm.nih.gov) and the taxonomic validity and lineage status of fungal groups and taxa identified by BLAST searches was determined based on information recovered from Mycobank (http://www.mycobank.org/). Multiple sequence alignments of newly produced sequences only (290 sequences) were computed in Clustal Omega [35] at www.ebi.ac.uk/Tools/msa/clustalo/, MAFFT [36] at www.ebi.ac.uk/Tools/msa/mafft/, MUSCLE [37] at www.ebi.ac.uk/Tools/msa/muscle/) and KALIGN [38] at www.ebi.ac.uk/Tools/msa/kalign/) under default conditions for DNA sequence alignments. The most common default options were for Clustal Omega: mBed-like clustering iteration = yes, max guide tree iterations = 1, max HMM iterations = 1, gap open penalty = 6, gap extension penalty = 1; MAFFT: tree rebuilding number = 1, guide tree output = on, max number of iterations = 0, perform fast fourier transform = localpair, gap open penalty = 1.53, gap extension penalty = 0.123; MUSCLE: max number of iterations = 16, gap open penalty = -12.0, gap extension penalty = -1.0; KALIGN: gap open penalty = 11, gap extension penalty = 0.85). In addition, publically available sequences corresponding to the best BLAST matches were merged with the 290 new sequences (a total of 349 barcodes) and aligned in KALIGN under default settings. For each of the resulting alignments, the neighbour joining algorithm (NJ) was used in PAUP*4.0b10 for Windows [39] to infer phylogenies from distance matrixes calculated following distance corrections suggested by Modeltest [30]. The consistency index (CI), retention index (RI), tree length and the amount of phylogenetic signal versus noise (g_1_ statistics; [40]) were assessed in the same software. Finally, pairwise p-distance, Kimura-2-Parameter (K2P) [41] and General Time Reversible (GTR) model-corrected genetic distances were computed in MEGA5 [42]. Automatic barcode gap discovery (ABGD) was performed by importing distance matrixes into the web version of ABGD [43] (http://wwwabi.snv.jussieu.fr/public/abgd/abgdweb.html) for identification of the barcode gap and assignment of sequence clusters into hypothetical OTUs. In ABGD, ITS sequences are regrouped into hypothetical species based on the barcode gap principle [43]. Briefly, following pairwise sequence comparisons, it is expected that genetic distances among sequences within a hypothetically genetically homogenous species are lower compared to genetic distances recovered from pairwise comparisons among biologically distinct species. The range of genetic distances in between, i.e. the barcode gap, is not present in the matrix and can be considered as a set of *a priori* threshold values for the delineation of genetically distinct taxa [43]. The aforementioned alignments were additionally used to compute intraspecific K2P-corrected distances.

### Model selection & Bayesian phylogenetic inference

A Bayesian phylogenetic hypothesis was inferred from the global dataset (a total of 349 sequences). Hierarchical Likelihood Ratio Tests (hLRTs) were run in Modeltest v3.7 [30] to identify the best-fitting model and fix the parameters (gamma distribution, proportion of invariable sites, transition-transversion ratio) during tree searches for Bayesian Inference (BI) implemented in MrBayes v3.1.2 [44]. BI served to identify topologically statistically robust, reciprocally monophyletic groups of sequences, sitting on relatively long branches that may correspond to OTUs. BI was conducted for 2,000,000 generations of two parallel runs of four chains each, starting from a random tree and sampling every 1,000 generations. The convergence of the parameter estimates was confirmed by plotting likelihood values against generation time in Tracer v1.5 (Available at: http://beast.bio.ed.ac.uk/Tracer).

## Results

### Sequence analysis and characterization of marine-derived fungal isolates

**Fig 2:**
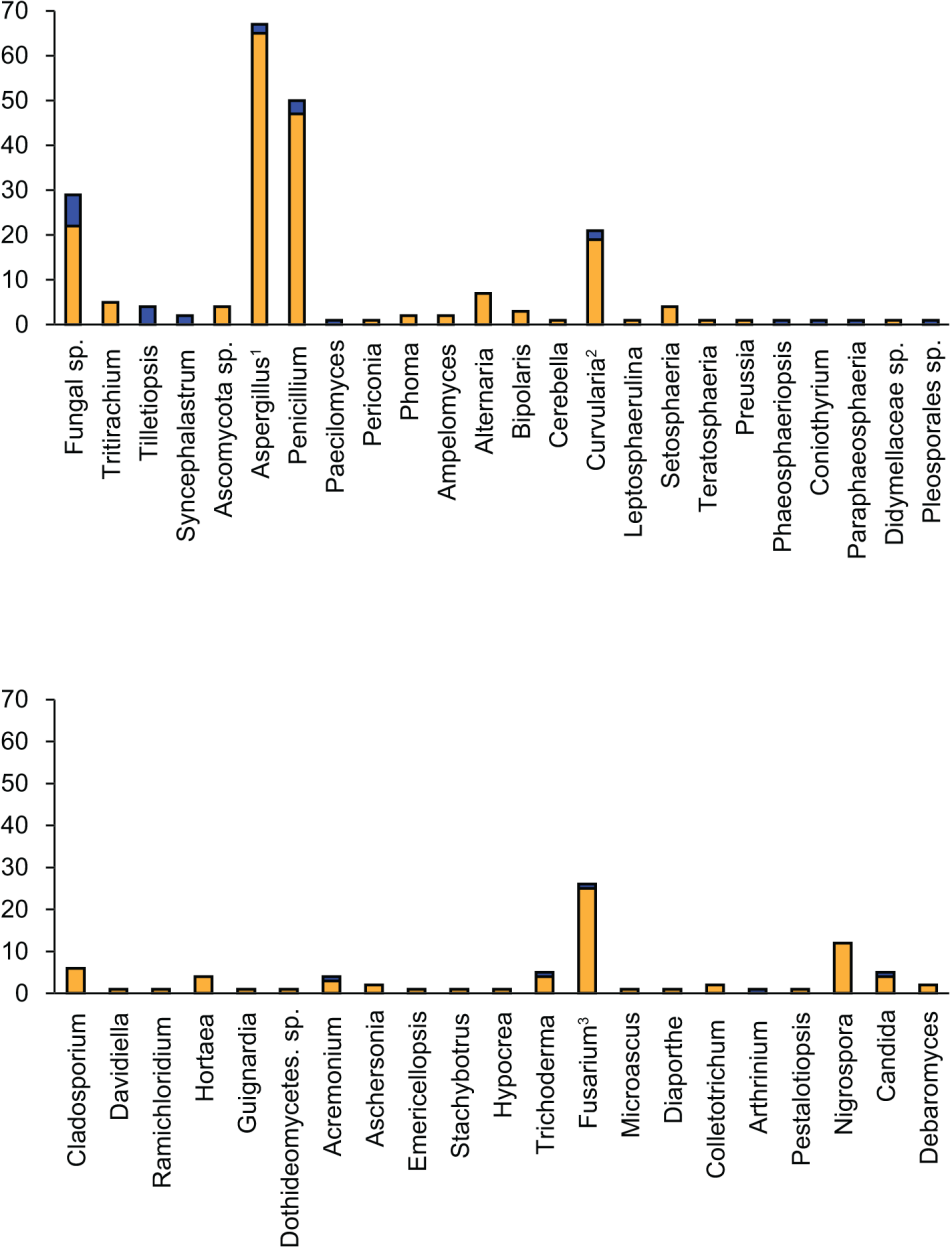
BLAST search results. Genus-level frequency distributions of isolates based on best BLAST matches for the barcodes produced in this study. The blue part of each bar corresponds to isolates that we classified as ‘unknown’ based on low percentage sequence identity with the best BLAST match and branch length in the phylogenetic tree (Figs 4, 5, 6 and 7). ^1^Isolates with best BLAST match with the teleomorphs *Eurotiales* or *Neosartorya* are included. ^2^Isolates with best BLAST match with the teleomorph *Cochliobolus* are included. ^3^Isolates with best BLAST match with the teleomorph *Gibberella* are included.

**Fig 3:**
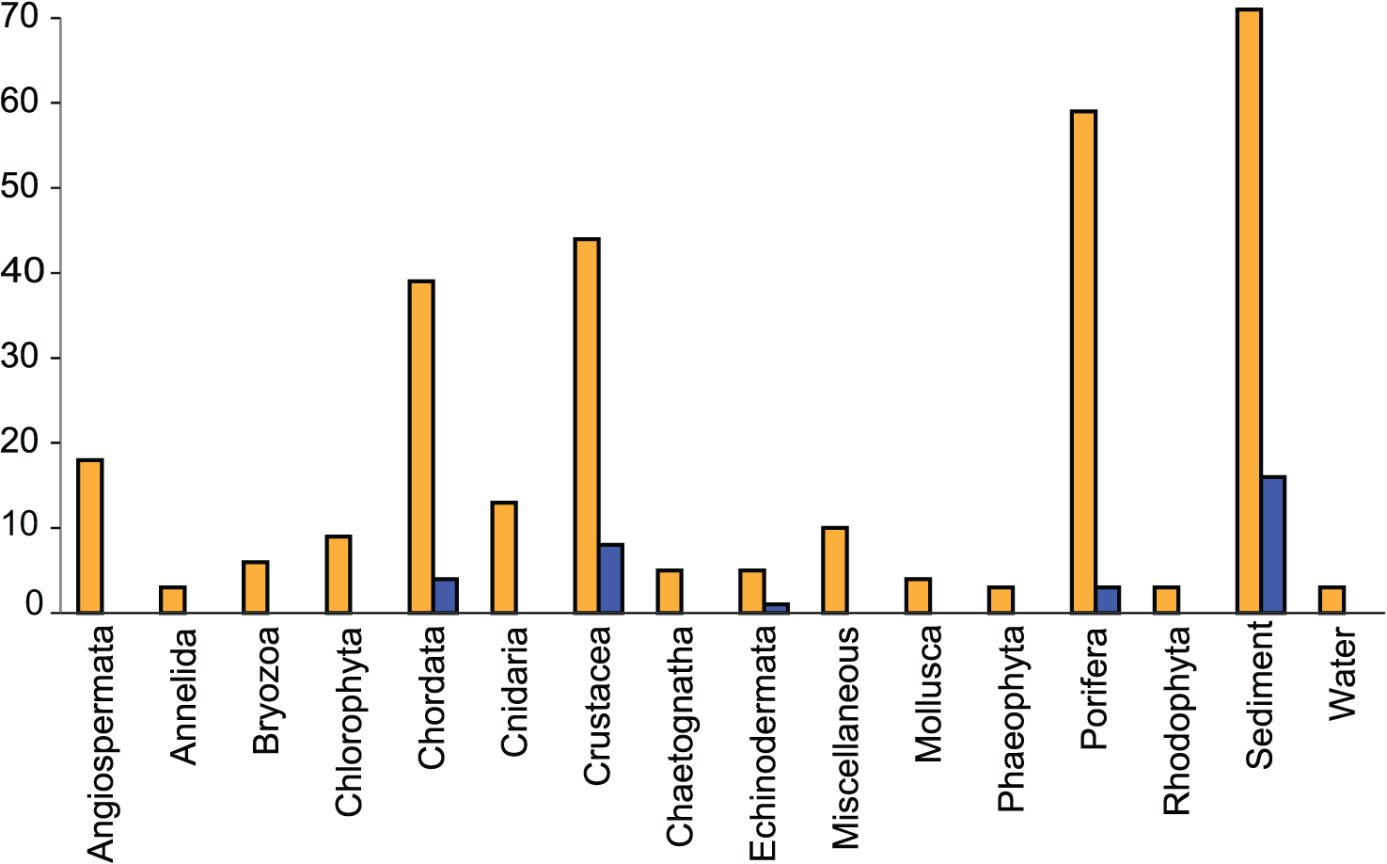
Isolate sources. The number of isolates per source type is shown as orange bars. The number of unknown isolates per source type is shown as blue bars.

**Fig 4:**
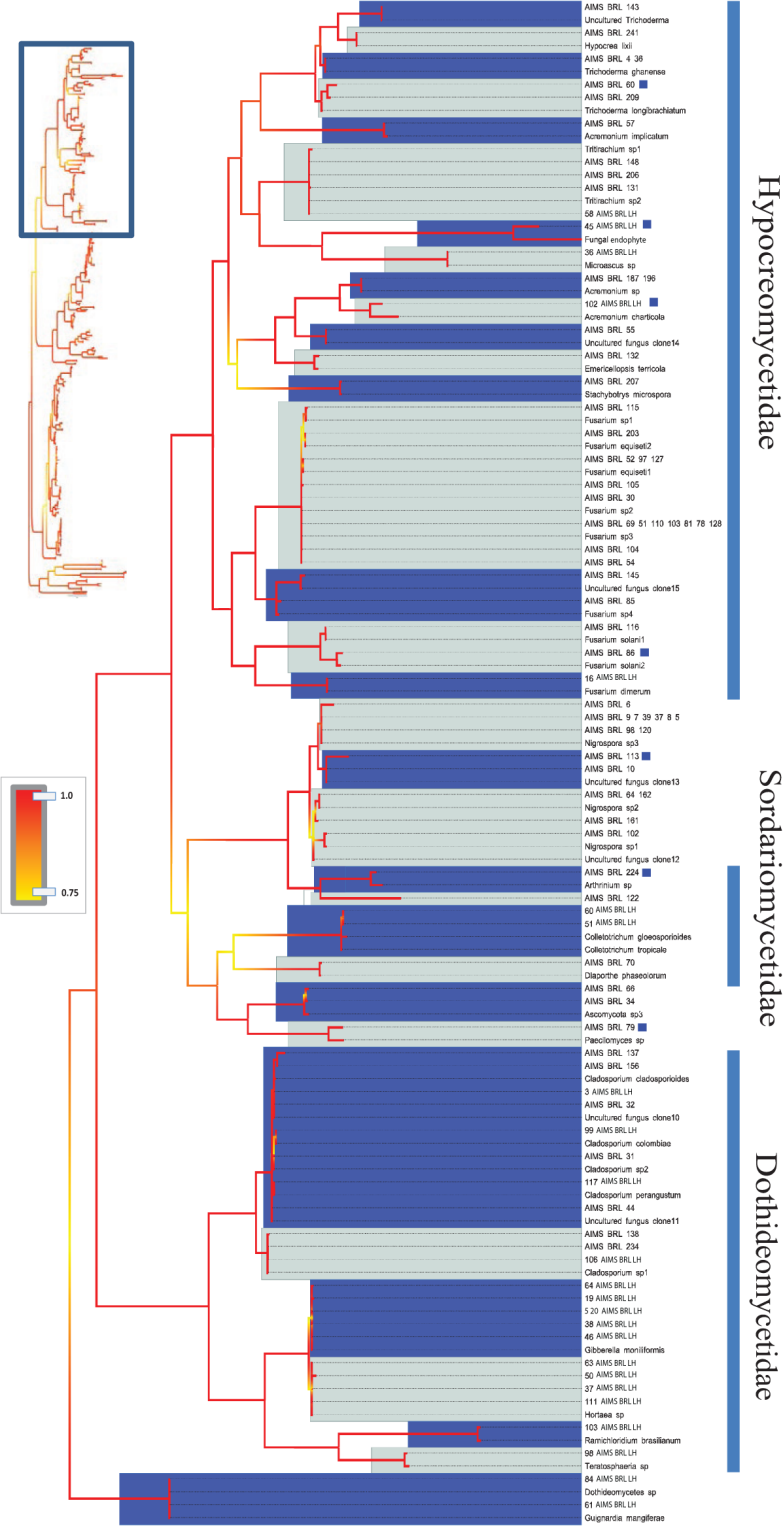
Bayesian phylogenetic hypothesis inferred from ITS barcodes produced in this study and corresponding best BLAST results. Subsection of tree focused on Hypocreomycetidae, Sordariomycetidae and Dothideomycetidae isolates. Alternating dark blue and grey blocks are intended to visually differentiate reciprocally monophyletic OTUs. Yellow-to-red colour gradient denotes posterior probability support throughout the topology. Blue boxes denote unknown isolates.

**Fig 5:**
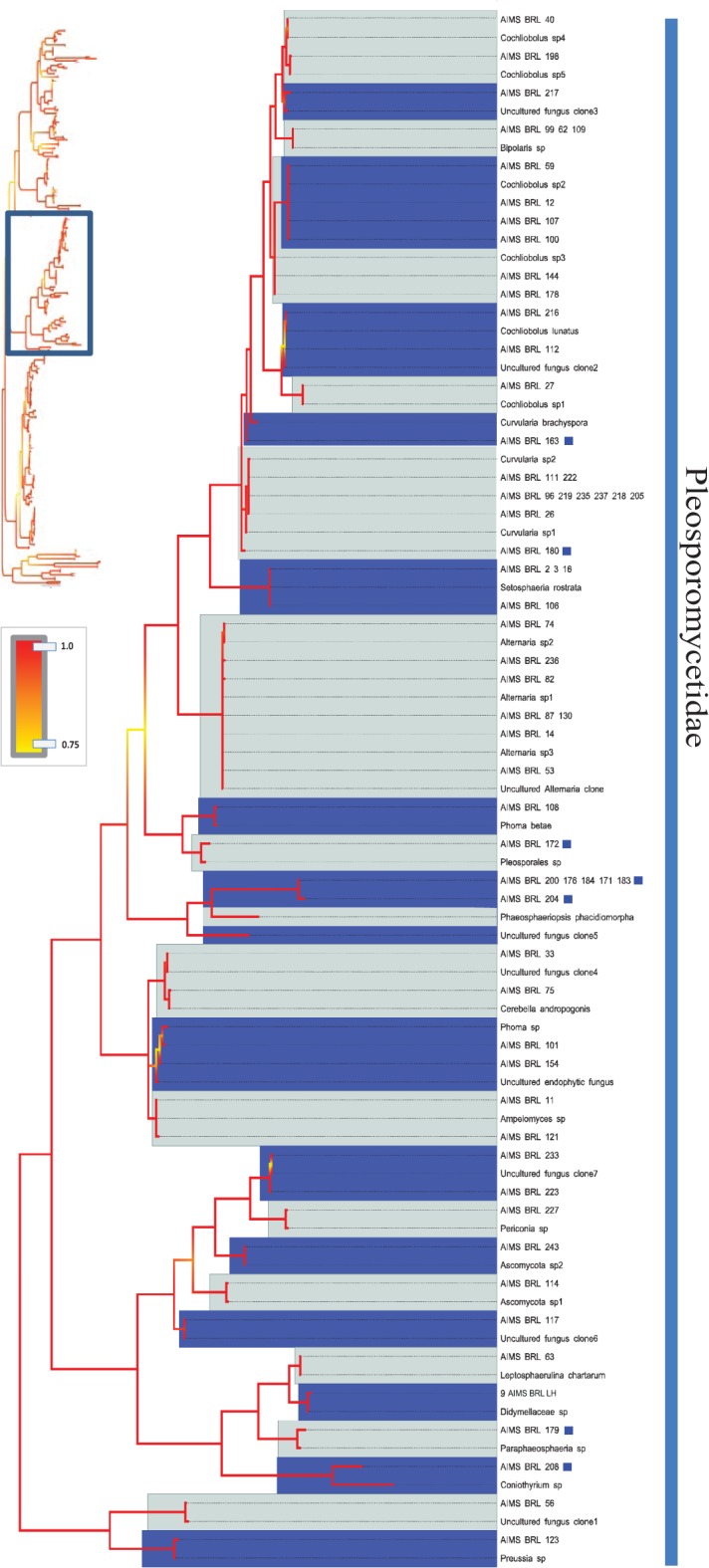
Bayesian phylogenetic hypothesis inferred from ITS barcodes produced in this study and corresponding best BLAST results. Subsection of tree focused on Pleosporomycetidae isolates. Alternating dark blue and grey blocks are intended to visually differentiate reciprocally monophyletic OTUs. Yellow-to-red colour gradient denotes posterior probability support throughout the topology. Blue boxes denote unknown isolates.

**Fig 6:**
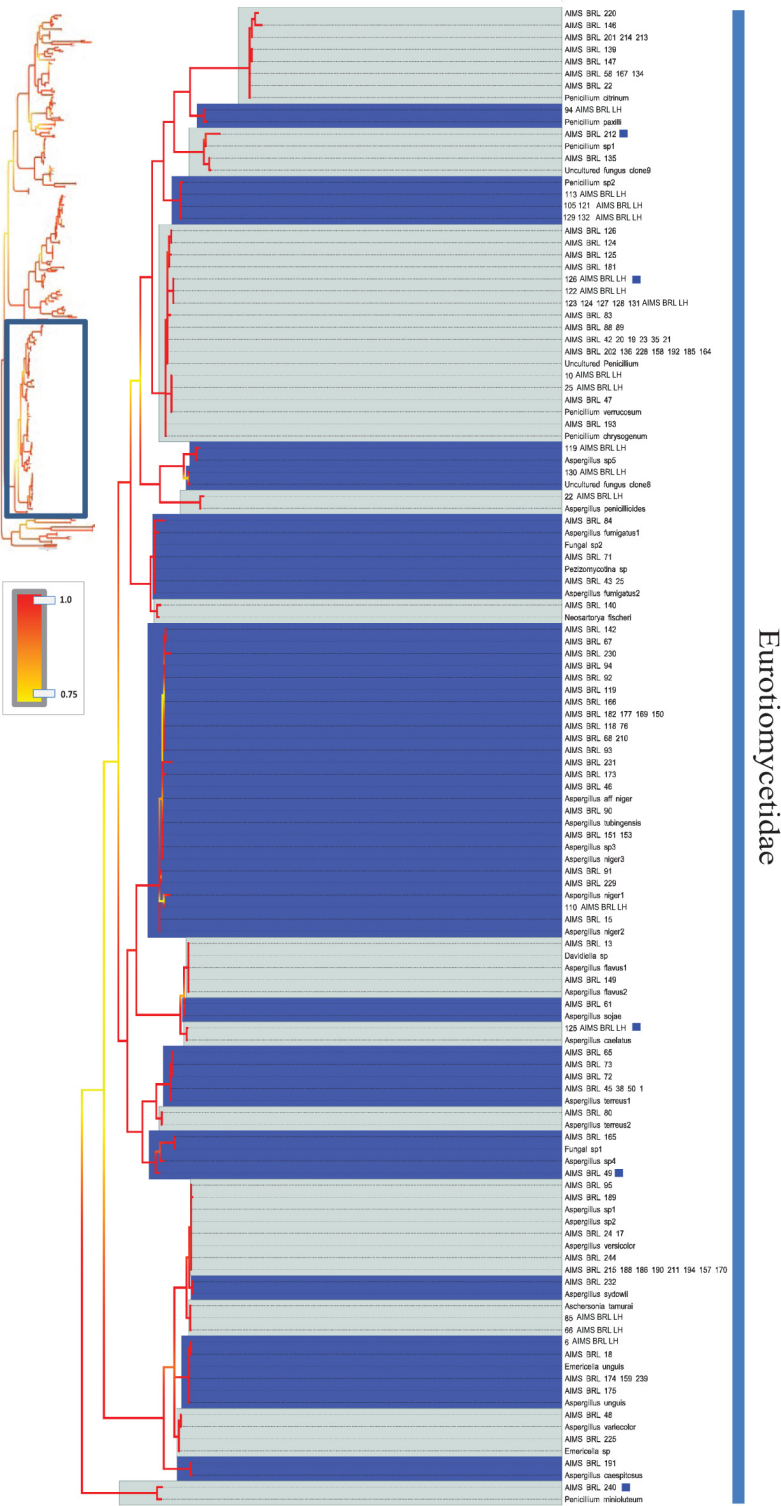
Bayesian phylogenetic hypothesis inferred from ITS barcodes produced in this study and corresponding best BLAST results. Subsection of tree focused on Eurotiomycetidae isolates. Alternating dark blue and grey blocks are intended to visually differentiate reciprocally monophyletic OTUs. Yellow-to-red colour gradient denotes posterior probability support throughout the topology. Blue boxes denote unknown isolates.

**Fig 7:**
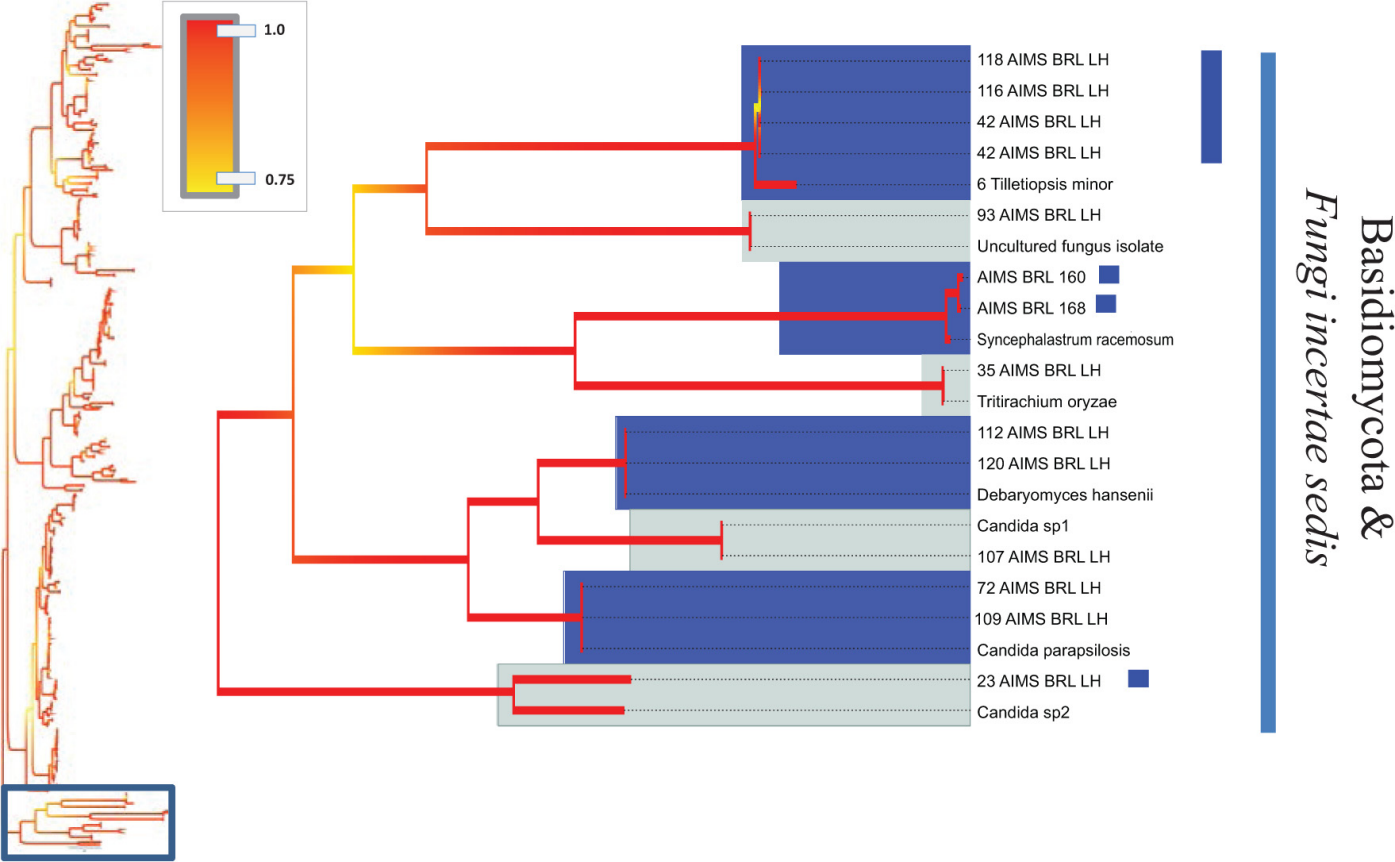
Bayesian phylogenetic hypothesis inferred from ITS barcodes produced in this study and corresponding best BLAST results. Subsection of tree focused on Basidiomycota and *Fungi incertae sedis* isolates. Alternating dark blue and grey blocks are intended to visually differentiate reciprocally monophyletic OTUs. Yellow-to-red colour gradient denotes posterior probability support throughout the topology. Blue boxes denote unknown isolates.

The barcodes produced from 290 viable fungal isolates corresponded to 214 unique sequences (see Table 1 for sequence and alignment statistics). An overview of the best BLAST matches returned for each isolate’s ITS sequence is presented in S2 Table. The majority of the barcodes were successfully resolved at the species or genus levels (see below), and 50 unique ITS sequences corresponded to uncultured fungus clones. Interestingly, a combination of tree topology and BLAST search results (<98% sequence identity) suggested that 26 unique ITS barcodes (a total of 30 isolates) differed significantly from the most closely related sequences deposited in NCBI. Since many already described fungal species lack barcoding information, we refer to these 26 OTUs as unknown rather than new OTUs, strains or species (Figs 2, 3, 4, 5, 6 and 7). Fig 2 presents an overview of the taxonomic frequency distribution of isolates based on the best BLAST match of their respective ITS sequences. For each group, the number of best BLAST matches that were classified as unknown based on the criteria above is indicated.

**Table 1:** Sequence and alignment statistics. Pu, parsimony uninformative characters; pi, parsimony informative characters;-g_1_ statistics, the amount of phylogenetic signal versus noise; model, model of molecular evolution inferred by Modeltest; I, proportion of invariable sites; G, gamma distribution shape parameter alpha;-lnL, maximum likelihood tree score; tree length, the minimum number of substitutions over all sites for a given topology; ME, Minimum evolution score; CI, consistency index (the amount of homoplasy given the data; equals 1 when there is no homoplasy); RI, retention index (the amount of synapomorphy on the tree; increased values from 0 to 1 suggest increased evidence of grouping).

Alignment	# sequences	speed	length	pu	pi	-g_1_	model	I	G	-lnL	Tree length	ME scores	CI	RI
**CLUSTAL-O**	290	21”	740	52	569	-0.21	GTR	0.109	0.822	30452.8574	7850	23.004	0.195	0.843
**MAFFT**	290	49”	1183	195	712	-0.16	GTR	0.093	0.816	24709.5938	5967	13.029	0.322	0.87
**MUSCLE**	290	3’58”	1365	291	715	-0.22	GTR	0.079	0.807	31833.6211	7508	13.741	0.276	0.813
**KALIGN**	290	8”	**1785**	290	**769**	-0.19	TN	0.092	0.830	24071.0117	**5281**	**12.092**	0.38	0.873
**KALIGN-BI**	349	11”	**1815**	126	**1061**	-0.17	TN	0.083	0.853	27039.1914	**5860**	**12.568**	0.37	0.89

Of the 290 isolates, 29 (10%) yielded best BLAST hits that were classified only as Fungal sp., of which 25 were recovered from clones. The majority of the remaining isolates were most closely related with Ascomycota, with the exception of 9 isolates (3.1%) that returned best matches related to Basidiomycota (genera *Tritirachium* and *Tilletiopsis*), and 2 isolates (0.7%) that returned best matches related to Zygomycota (genus *Syncephalastrum*). Of the Ascomycota isolates, 4 returned BLAST matches that were identified to that level only. Of the remaining isolates, 118 (40.7% of total) returned best BLAST matches related to the class Eurotiomycetes. All of these belonged to the subclass Eurotiomycetidae, order Eurotiales; genera *Aspergillus* and its sexual forms *Emericella* and *Neosartorya* (total 67 isolates), *Penicillium* (50 isolates, of which 20 were most closely related to clone sequences), and *Paecilomyces* (1 isolate).

The class Dothideomycetes included the best BLAST match for 63 isolates (21.7% of total). Of these, 49 (16.9% of total) belonged to a wide variety of genera in subclass Pleosporomycetidae, order Pleosporales (genera *Periconia*, *Phoma*, *Ampelomyces*, *Alternaria*, *Bipolaris*, *Cerebella*, *Curvularia* and its sexual form *Cochliobolus*, *Leptosphaerulina*, *Setosphaeria*, *Teratosphaeria*, *Preussia*, *Phaeosphaeriopsis*, *Coniothyrium*, *Paraphaeosphaeria*; Didymellaceae sp., and Pleosporales sp.). A further 12 belonged to the subclass Dothideomycetidae, order Capnodiales (genera *Cladosporium*, *Davidiella*, *Ramichloridium* and *Hortaea*); 1 belonged to the order Botryosphaeriales for which no subclass is defined (genus *Guignardia*); and 1 could not be more precisely identified (Dothideomycetes sp.).

The class Sordariomycetes included the best BLAST match for 58 isolates (20.0% of total). Of these, 40 belonged to the subclass Hypocreomycetidae, order Hypocreales (genera *Acremonium*, *Aschersonia*, *Emericellopsis*, *Stachybotrus*, *Hypocrea*, *Trichoderma*, *Fusarium* and its sexual form *Gibberella*); 1 isolate belonged to subclass Hypocreomycetidae, order Microascales (*Microascus*); 4 belonged to the subclass Sordariomycetidae (genera *Diaporthe*, *Colletotrichum*, and *Arthrinium*); and 1 belonged to the subclass Xylariomycetidae (genus *Pestalotiopsis*). The remaining 12 Sordariomycetes isolates returned BLAST matches belonging to the order Trichosphaeriales (genus *Nigrospora*), for which a subclass has not been defined.The class Saccharomycetes included the best BLAST match for 7 isolates (2.4% of total); all belonging to subclass Saccharomycetidae, order Saccharomycetales (genera *Candida* and *Debaromyces*). The majority of the strains were isolated from sediment, crustaceans and sponges (Fig 3), as could be expected since these sources were represented by the highest number of isolates.

Given the uneven number of ITS sequences recovered within each OTU we could not make global assessments on the levels of intraspecific divergence encountered. However, in cases where multiple sequences were available, K2P corrected intraspecific genetic distances ranged from 0.0073 in *Aspergillus terreus* to 0.018 in *Penicillium verrucosum*, with *Fusarium equiseti* having a value of 0.015. The taxonomic depth of the analysed isolates is illustrated in Figs 4, 5, 6 and 7.

### Multiple sequence alignment and evolutionary distance estimates

Table 1 presents alignment statistics for each of the tested alignment algorithms, performed online under default settings. Indels represent a common feature of intergenic spacers such as the ITS regions. Their presence and abundance is expected to differentially influence the speed and phylogenetic informativeness of each of the tested algorithms. Speed varied significantly between algorithms, with KALIGN and MUSCLE resulting in the shortest and longest calculation times, respectively. Calculation times for the CLUSTAL-O and MAFFT algorithms were nearly as fast as for KALIGN. The General Time Reversible substitution model (GTR; six relative rates, variable base frequencies) [45] together with substitution rate heterogeneity over the alignment sites modelled by two additional parameters, gamma distribution (G; shape parameter alpha) and proportion of invariant sites (I), appeared to fit the data best in all cases (Table 1) but KALIGN. In the letter case the simpler Tamura-Nei model (TN; variable base frequencies, equal transversion rates, variable transition rates) [46] with substitution rate heterogeneity over the sites modelled by G and I was selected. Two interesting trends emerged from the alignment statistics. These were an increase in alignment length and in the number of parsimoniously informative sites (PI) recovered, with CLUSTAL-O being the most conservative and KALIGN the most flexible in accommodating gaps (Table 1). However, independently of the number of PIs, tree reconstruction may not be reliable if the extent of homoplasy in the alignment is significant [47, 48]. In comparisons here, KALIGN demonstrated to be the most accurate. The latter algorithm revealed the lowest levels of homoplasy as suggested by the consistency index and was the one with the larger amount of synapomorphic characters, which facilitates the creation of clusters of sequences (Table 2). The alignments produced in this study are available as S1–S5 Files.

**Table 2:** Number of OTUs identified. The number of OTUs is reported given the alignment algorithm, the genetic distance calculation methodology and the *a priori* established primary intraspecific distance partitions in ABGD. p-distance, the proportion (p) of nucleotide sites at which two sequences being compared are different; K2P distance, Kimura’s 2-parameter distance correction which takes into account transitional and transversional substitutions while assuming equal nucleotide frequencies and invariable substitutional rates among sites; GTR, General Time Reversible evolutionary model based on six substitution rate parameters and 4 equilibrium base frequency parameters.

	p-distance	K2P	GTR
**CLUSTAL-O**	84	84	84
**MAFFT**	67	67	67
**MUSCLE**	82	82	78
**KALIGN**	81	83	83

### Genetic distance and inference of the number of OTUs

**Fig 8:**
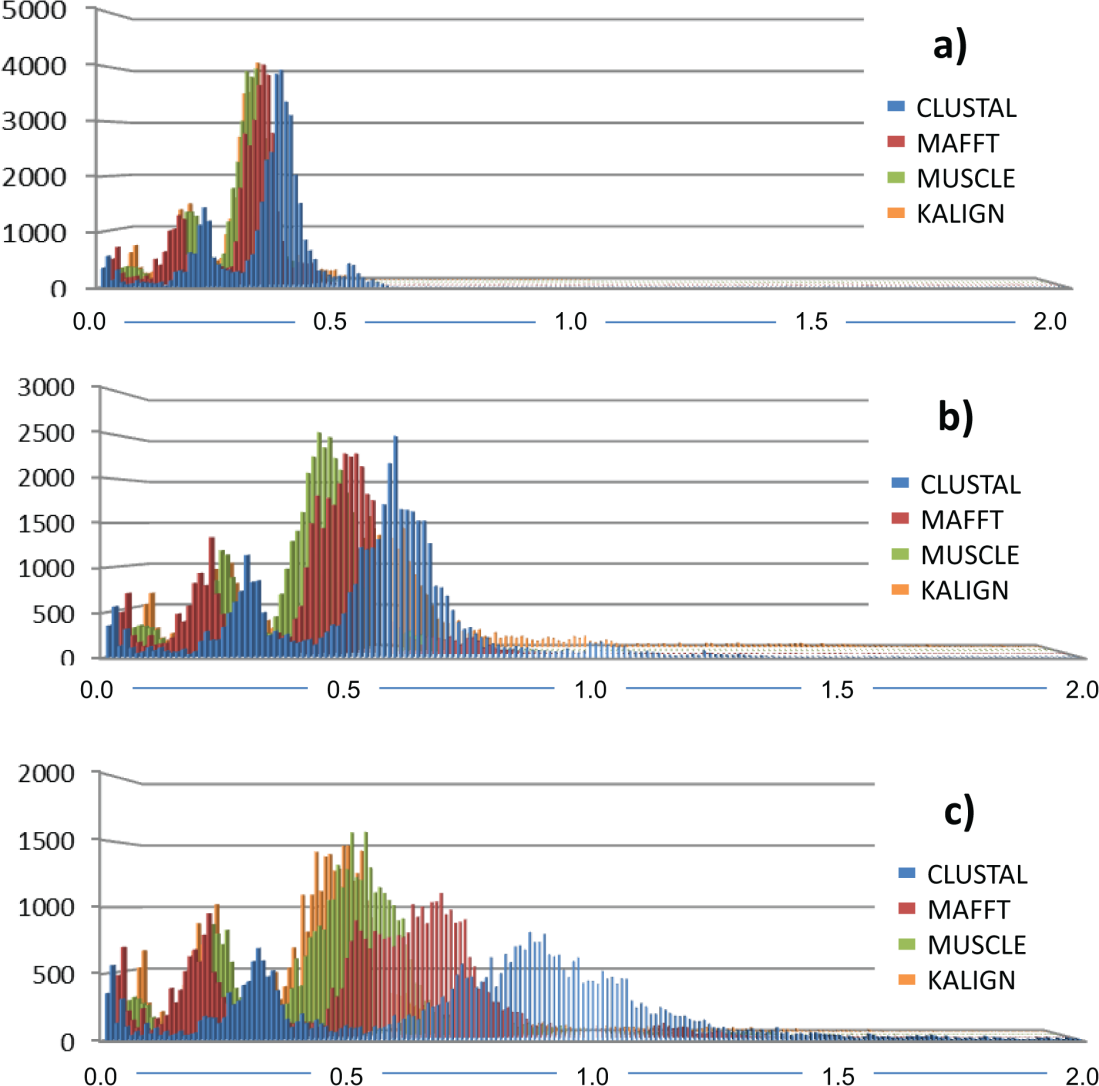
Frequency distribution of pairwise comparisons given the alignment. a) p-distance; b) K2P correction and c) GTR+I+G model to assess the influence of the alignment algorithm on the inferred genetic distance. Alignments were performed using default settings in MUSCLE, MAFFT, CLUSTAL and KALIGN.

The alignment algorithm significantly influenced the genetic distance calculation. The frequency distribution of pairwise comparisons was more conservative when the simpler *p*-distance calculation was applied to the dataset and increased with the increased number of parameters involved in the distance correction from K2P to the GTR model (Fig 8). Downstream analyses, such as calculation of the barcoding gap and the identification of the number of OTUs via the ABGD, were consequently affected (Table 3). The barcoding gap varied significantly given the alignment algorithm and the genetic distance correction (S1 Fig, S2 Fig, S3 Fig and S4 Fig). The number of OTUs defined by ABGD varied consequently from 67 to 84 given the *a priori* intraspecific genetic distance matrix (Table 2, S1 Fig, S2 Fig, S3 Fig and S4 Fig for ABGD statistics). *P*-distance, K2P and GTR distance corrections always delivered the same number of OTUs with the same alignment algorithm in two cases (84 and 67 for CLUSTAL-O and MAFFT respectively). In contrast, for the MUSCLE alignment, the *P*- and K2P distances produced a larger number of OTUs (82) than the GTR corrected distance (78).

**Table 3:** ABGD analysis statistics. The number of operational taxonomic units is reported given the alignment algorithm, the genetic distance calculation methodology and the *a priori* established recursive intraspecific distance partitions in ABGD as a function of the prior limit between intra- and inter-specific divergence. p-distance, the proportion (p) of nucleotide sites at which two sequences being compared are different; K2P distance, Kimura’s 2-parameter distance correction which takes into account transitional and transversional substitutions while assuming equal nucleotide frequencies and invariable substitutional rates among sites; TN, Tamura-Nei model based on variable base frequencies, equal transversion rates and variable transition rates.

		CLUSTAL			MAFFT			MUSCLE		KALIGN			
Partitions	prior distance	*p*-distance	K2P	TN	*p*-distance	K2P	TN	*p*-distance	k2p	TN	*p*-distance	K2P	TN
**1**	0.001	134	134	133	118	120	116	137	137	137	124	124	124
**2**	0.0017	127	126	122	112	112	106	136	135	136	124	124	124
**3**	0.0029	108	107	107	96	96	95	122	121	122	100	100	100
**4**	0.0049	106	106	104	93	93	94	117	117	117	97	97	98
**5**	0.0084	101	101	99	90	90	90	114	114	114	96	96	96
**6**	0.0143	96	96	96	85	85	78	100	100	100	93	93	93
**7**	0.0243	90	90	90	68	68	68	94	94	94	81	83	83
**8**	0.0414	1	1	64	67	67	67	1	1	1	1	3	1
**9**	0.0705	1	1	2	1	1	2	1	1	1	1	3	1
**10**	0.12	1	1	2	1	1	2	1	1	1	1	3	1

## Discussion

The diversity, phylogeny and ecology of marine fungi are vastly under-explored, at least partly due to the relatively few isolates available in culture [4]. A major outcome of this study is the presentation of 290 barcoded, marine-derived, culturable isolates that can be further studied to improve the collective understanding of fungal genomics, evolutionary relationships, physiology and production of bioactive compounds. This study also reports multiple previously unknown fungal lineages derived from several marine sources and the first cultured strains of several lineages previously represented only by fungal clones.

All isolates analyzed in this study have demonstrated salt tolerance, as they were isolated and cultured from marine samples and, in almost all cases, by using seawater-based media. More than half of the strains were isolated from samples collected far (> 20 km) from the nearest land and terrigenous influence, reducing the likelihood that they represent transient marine fungi. However, as each strain’s salt requirements were not characterized in detail, it is not possible to claim they are obligate marine fungi. Hence, the term ‘marine-derived fungi’ is used throughout this paper.

Morphological identification of fungal isolates at the species level is rarely conclusive as many fungi do not develop traditional diagnostic features, such as reproductive characters, under laboratory conditions [23]. Given the vague species boundaries amongst cultivated fungi, active bio-compounds are often reported from isolates without accurate taxonomic assessment of the isolate itself [23, 49]. Consequently, there is a need for development of DNA-based, standardized procedures for differentiating strains or monophyletic lineages, independently of a specific species concept [15]. Several authors have argued that DNA barcoding and comparison against sequences deposited in public databases represents a promising alternative for the identification of fungal OTUs [22, 23, 49].

### Phylogenetic diversity as revealed by ITS barcodes

The extent of phylogenetic coverage and the degree of variation found in this study are astonishing given the relatively small number of specimens analyzed. BLAST searches and phylogenetic reconstructions showed that the majority of the isolates belonged to the Ascomycota subclasses Eurotiomycetidae, Hypocreomycetidae, Sordariomcyetidae, Pleosporomycetidae, and Dothideomycetidae. Other Ascomycota subclasses that were represented include Xylariomycetidae and Saccharomycetidae, as well as some taxa whose exact phylogenetic lineage is not yet properly resolved (e.g. *Nigrospora*). The phylum Basidiomocyta was represented by isolates affiliated with the genera *Tritirachium* and *Tilletiopsis*. Some previous reports have claimed that basidiomycetes are largely excluded from aquatic habitats [2], however more recent studies have showed that they are relatively frequently retrieved in molecular studies of fungi in marine environments [4]. Future characterization of the salt tolerance of the Basidiomycota isolates identified in this study would be of interest, as Shearer and coworkers reported that only 10 of 465 known species of marine fungi are basidiomycetes [2]. Two isolates, recovered from sponge and intertidal sediment, returned best BLAST matches most closely related to the zygomycete *Syncephalastrum racemosum* (S2 Table). We classified these isolates as ‘unknown’ due to the low % sequence identity with database sequences (see below). Their further characterization is of interest since zygomycetes were reported as mostly absent from aquatic habitats except when using dilution plating of sediments and water [2].

The analysis revealed 26 unknown OTUs and an additional 50 isolates corresponding to previously uncultured, unidentified fungal clones. Some of these isolates may represent already described fungal species, as available molecular information is particularly limited for marine fungi [50] with published ITS barcodes available for only approximately one fifth of the ca. 100,000 described fungal species [51]. We therefore refer to the 26 OTUs as unknown OTUs or unknown fungal lineages rather than new species (Figs 2, 3, 4, 5, 6 and 7). Future efforts to genome sequence or barcode existing fungal type strains are needed to determine whether the unknown OTUs described in this study represent novel species or simply new strains of already described species. The results from this relatively small subsample of 290 fungal isolates suggest that the remaining nearly 1,500 fungal isolates in the AIMS Bioresources Library are likely to include further and considerable unknown or undescribed fungal diversity, emphasizing the value of such collections in exploring marine diversity and inherent bioactivity.

All known genera found in this study correspond to fungi previously reported in the marine natural products literature as sources of novel chemistry and bioactivity [7, 52, 53]. The majority of the 26 unknown OTUs were recovered from sediment samples, followed by Crustacea and Porifera, in line with the relative sampling effort associated with these sources (Fig 3). Sediments are a well-established source of marine fungi presumed to be involved in decomposition of detritus [54, 55]. However, sediments are also well known to contain particulate matter of terrestrial origin transported into and within the marine environment due to runoff from land and sediment transport processes [56]. Thus, fungi isolated from marine sediments may either represent truly marine lineages present at the time of culture, or terrestrial lineages washed into the marine environment via runoff. The majority of specimens contained representatives within *Aspergillus*, *Fusarium* and *Penicillium*. The dominance of these three most abundant genera (Fig 2) may represent sampling bias that would not be replicated in direct molecular surveys, especially in samples from sediments which may accumulate spores not originating from the marine environment *per se*. These genera are however generally detected in studies of fungi in marine environments (e.g. [4]), and *Aspergillus sydowii* is a demonstrated pathogen of sea fans [57]. Further, as food-particle retention by filter feeders such as sponges is known to be extremely selective and highly efficient at least in some species [58], it is unlikely that spores of terrigenous origin would passively accumulate within their tissues over time. Indeed, some sponges are adversely impacted by exposure to fine particles of terrigenous origin [59]. Nevertheless, the only way to determine if the isolates described in this study are obligate or transient marine fungi would be to test their growth under different salinities.

### Analytical considerations

#### ITS barcodes

In recent decades the ITS1–5.8S-ITS2 region of the nuclear ribosomal gene operon has been successfully used for species delineation and analysis of fungal diversity [19]. However, caution has been recommended when using universal primers targeting this region in environmental samples via NGS techniques [20, 60]. The ITS region occurs in multiple tandem repeats on one or more chromosomes allowing easy PCR amplification from minute amounts of DNA. The marker is known to possess a variable number of paralogous copies, in some cases not yet homogenized by concerted evolution [61], that may interfere severely with phylogenetic inference or species identification especially when population-level divergence has occurred or shallow diversification events are targeted [62, 63].

Taxonomic incongruences were observed when isolates with affinity to Ascomycota yeasts, such as *Candida* spp. and *Debaryomyces hansenii*, were recovered within the Basidiomycota/*Fungi incertae sedis* clade (Fig 7). The barcodes produced in this study showed no ambiguous positions (i.e. double peaks) and low intraspecific variation suggesting that homogenization of the excessive paralogous copies via concerted evolution may have taken place. It is therefore plausible that several complexities may have contributed to inaccuracy. First, it is known that the marker’s intraspecific variability may vary significantly within groups of the fungal kingdom and its implications on fungal taxonomy and phylogenetics are still debated [64]. Second, the identification accuracy of new strains and sequences is limited if the coverage of the reference database is not sufficiently complete [65]. In addition, a considerable portion of deposited fungal barcodes (ca. 20%) are suspected to be incorrectly annotated to species level [51, 65, 66]. However, since multiple strains were affected and their closest database matches differed, the latter two arguments are less likely to explain the taxonomic incongruences in Fig 7. The observed topological anomaly illustrates the usefulness and the limitation of DNA barcodes; while they are suitable for tentative identification of isolates they have limited value for establishing phylogenetic or evolutionary relationships. As the phylogenetic resolution power of a barcoding marker saturates with increasing number of sequences, accurate phylogenetic reconstructions in fungi require sequencing of multiple genes or complete genomes. Although this is achievable for taxa represented by isolates, it is beyond the scope of the current study.

#### Automated Barcode Gap Discovery

The influence of the multiple sequence alignment algorithm and evolutionary distance estimator in automatic OTU identification via ABGD was significant. Several quantitative approaches integrating tree-based methodologies (e.g. neighbor joining; NJ) or threshold-based automatic scoring of the number of OTUs are commonly used for barcode analysis [67–69]. Where clustering methods are applied, two preprocessing steps, namely sequence alignment and genetic distance evaluation, are essential for the accuracy of the barcoding results. The process is achievable for small datasets and when gene-coding barcodes, such as the Cytochrome oxidase I (COI), are aligned. Analysis typically involves manual editing steps such as translation of the sequences to amino-acids, alignment and *de novo* translation to nucleotides, and trimming [70]. Erroneous local alignment on the other hand is more likely to occur for DNA regions characterized by high rates of change and secondary structures such as the rRNA gene spacers or the 16S rRNA gene [71]. Any inaccurate alignments that remain after manual correction will influence directly the genetic distance calculation, the phylogeny and consequently falsify the choice of the threshold used in automatic procedures for OTUs delineation. The aforementioned effects are obviously dramatic when barcodes of unequal length, deriving from large environmental datasets of broad phylogenetic coverage and variable levels of molecular evolution, are analyzed [72].

ABGD partitions barcodes into OTUs based on a threshold [43] and has been proposed as a valuable tool for computationally efficient OTU prediction from large sequence datasets, uncharacterized groups and environmental data [17, 43, 73]. This study demonstrates that the choice and accuracy of the alignment algorithm has significant impact on downstream data analyses such as automated OTU delineation. For example, following sequence alignment by CLUSTAL-O, ABGD delivered 84 OTUs independently of the genetic distance correction. This result was comparable to those obtained with MUSCLE and KALIGN, while MAFFT was the most conservative algorithm producing only 67 OTUs (Table 2). Barcoding regions such as the ITS1–5.8S-ITS2 operon or the 16S rRNA gene embody secondary structure peculiarities and unequal substitution rates distributed throughout their length. Sequence alignment algorithms will consequently perform differently and according to their ability of incorporating secondary structure information in the alignment process.

## Conclusions

This study represents a substantial step towards understanding the diversity, phylogeny, and ecology of marine fungi, which is currently vastly underexplored. It has also facilitated future detailed studies of marine fungal biology and ecology by contributing 290 barcoded viable marine-derived isolates available for further characterization including full-genome sequencing and other—omics based approaches. In addition, these viable cultures represent an important future resource for phylogeny-directed biodiscovery. Finally, our analytical approaches have further contributed to the development of standardized barcoding protocol pipelines to determine unambiguous fungal OTUs, which will facilitate future studies of fungal genomics and physiology.

## Supporting Information

S1 File 290 sequences Clustal alignment. (TXT)

S2 File 290 sequences Kalign alignment. (TXT)

S3 File 290 sequences Mafft alignment. (TXT)

S4 File 290 sequences Muscle alignment. (TXT)

S5 File 349 sequences Kalign alignment. (TXT)

S1 Fig ABGD statistics based on genetic distances inferred from sequence alignment performed in CLUSTAL OMEGA at http://www.ebi.ac.uk/Tools/msa/clustalo/. Left column focuses on the Barcode gap zone (arrows) calculated from p-distance, K2P correction and the TN+G model respectively. The same pairwise comparisons are ranked in the middle column to visualize local slopes corresponding to the barcode gap zone. On the right column, grey dots denote the number of distinct groups (initial partitions) inferred from prior intraspecific divergence; red dots indicate the number of recursive partitions i.e. finer partitions until there is no further partitioning of the data.
(EPS)

S2 Fig ABGD statistics based on genetic distances inferred from sequence alignment performed in MAFFT at http://www.ebi.ac.uk/Tools/msa/mafft/. Left column focuses on the Barcode gap zone (arrows) calculated from p-distance, K2P correction and the TN+G model respectively. The same pairwise comparisons are ranked in the middle column to visualize local slopes corresponding to the barcode gap zone. On the right column, grey dots denote the number of distinct groups (initial partitions) inferred from prior intraspecific divergence; red dots indicate the number of recursive partitions i.e. finer partitions until there is no further partitioning of the data.
(EPS)

S3 Fig ABGD statistics based on genetic distances inferred from sequence alignment performed in MUSCLE at http://www.ebi.ac.uk/Tools/msa/muscle/. Left column focuses on the Barcode gap zone (arrows) calculated from p-distance, K2P correction and the TN+G model respectively. The same pairwise comparisons are ranked in the middle column to visualize local slopes corresponding to the barcode gap zone. On the right column, grey dots denote the number of distinct groups (initial partitions) inferred from prior intraspecific divergence; red dots indicate the number of recursive partitions i.e. finer partitions until there is no further partitioning of the data.
(EPS)

S4 Fig ABGD statistics based on genetic distances inferred from sequence alignment performed in KALIGN at http://www.ebi.ac.uk/Tools/msa/kalign/. Left column focuses on the Barcode gap zone (arrows) calculated from p-distance, K2P correction and the TN+G model respectively. The same pairwise comparisons are ranked in the middle column to visualize local slopes corresponding to the barcode gap zone. On the right column, grey dots denote the number of distinct groups (initial partitions) inferred from prior intraspecific divergence; red dots indicate the number of recursive partitions i.e. finer partitions until there is no further partitioning of the data.
(EPS)

S1 Table Material information. Source material, collection location, jurisdiction, distance to nearest landmass or island, and salinity condition of inshore sites of analyzed fungal isolates.
(DOCX)

S2 Table BLAST results. Accession numbers of ITS barcode sequences, their best BLAST hit, and percentage sequence identity.
(DOCX)
