# Supplementary material for: Diversity of Marine-Derived Fungal Cultures Exposed by DNA Barcodes: The Algorithm Matters
Source: PLoS One. 2015 Aug 26;10(8):e0136130. doi: 10.1371/journal.pone.0136130 (PMC4550264; doi:10.1371/journal.pone.0136130)
Supplement: S1 Table — Source material, collection location, jurisdiction, distance to nearest landmass or island, and salinity condition of inshore sites of analyzed fungal isolates. (DOCX) [file pone.0136130.s010.docx]

Table S1: **Material information.** Source material, collection location, jurisdiction, distance to nearest landmass or island, and salinity condition of inshore sites of analysed fungal isolates.

| Isolate label | Source phylum | Latitude | Longitude | Jurisdiction | Nearest land type | km to land | Description | Salinity | AIMS BRL nr |
| --- | --- | --- | --- | --- | --- | --- | --- | --- | --- |
| BRL-1 | Angiospermata | 19 22.00 S | 147 05.00 E | QLD | Mainland | 0 | Mangroves (establishing) | Full | 57462 |
| BRL-2 | Angiospermata | 19 22.00 S | 147 05.00 E | QLD | Mainland | 0 | Mangroves (establishing) | Full | 57463 |
| BRL-3 | Angiospermata | 19 22.00 S | 147 05.00 E | QLD | Mainland | 0 | Mangroves (establishing) | Full | 57466 |
| BRL-4 | Angiospermata | 19 22.00 S | 147 05.00 E | QLD | Mainland | 0 | Mangroves (establishing) | Full | 57481 |
| BRL-5 | Angiospermata | 19 22.00 S | 147 05.00 E | QLD | Mainland | 0 | Mangroves (establishing) | Full | 57482 |
| BRL-6 | Angiospermata | 19 22.00 S | 147 05.00 E | QLD | Mainland | 0 | Mangroves (establishing) | Full | 57483 |
| BRL-7 | Angiospermata | 19 22.00 S | 147 05.00 E | QLD | Mainland | 0 | Mangroves (establishing) | Full | 57485 |
| BRL-8 | Angiospermata | 19 22.00 S | 147 05.00 E | QLD | Mainland | 0 | Mangroves (establishing) | Full | 57515 |
| BRL-9 | Angiospermata | 19 22.00 S | 147 05.00 E | QLD | Mainland | 0 | Mangroves (establishing) | Full | 57516 |
| BRL-10 | Angiospermata | 19 22.00 S | 147 05.00 E | QLD | Mainland | 0 | Mangroves (establishing) | Full | 57517 |
| BRL-11 | Angiospermata | 19 22.00 S | 147 05.00 E | QLD | Mainland | 0 | Mangroves (establishing) | Full | 57518 |
| BRL-12 | Angiospermata | 19 22.00 S | 147 05.00 E | QLD | Mainland | 0 | Mangroves (establishing) | Full | 57523 |
| BRL-13 | Angiospermata | 19 22.00 S | 147 05.00 E | QLD | Mainland | 0 | Mangroves (establishing) | Full | 57524 |
| BRL-14 | Angiospermata | 19 22.00 S | 147 05.00 E | QLD | Mainland | 0 | Mangroves (establishing) | Full | 57534 |
| BRL-15 | Angiospermata | 19 22.00 S | 147 05.00 E | QLD | Mainland | 0 | Mangroves (establishing) | Full | 57557 |
| BRL-16 | Angiospermata | 19 22.00 S | 147 05.00 E | QLD | Mainland | 0 | Mangroves (establishing) | Full | 57573 |
| BRL-17 | Angiospermata | 19 22.00 S | 147 05.00 E | QLD | Mainland | 0 | Mangroves (establishing) | Full | 57604 |
| BRL-18 | Angiospermata | 19 22.00 S | 147 05.00 E | QLD | Mainland | 0 | Mangroves (establishing) | Full | 57607 |
| BRL-19 | Annelida | 21 29.41 S | 151 23.26 E | QLD | Island | 91 |  |  | 54836 |
| BRL-20 | Annelida | 21 04.30 S | 151 36.20 E | CWLTH | Island | 138 |  |  | 54840 |
| BRL-21 | Annelida | 21 29.41 S | 151 23.26 E | QLD | Island | 91 |  |  | 54849 |
| BRL-22 | Bryozoa | 19 10.70 S | 117 42.18 E | CWLTH | Mainland | 138 |  |  | 51616 |
| BRL-23 | Bryozoa | 20 35.17 S | 117 16.26 E | CWLTH | Island | 8 |  |  | 51648 |
| BRL-24 | Bryozoa | 20 35.17 S | 117 16.26 E | CWLTH | Island | 8 |  |  | 51649 |
| BRL-25 | Bryozoa | 20 27.97 S | 117 17.77 E | CWLTH | Island | 16 |  |  | 51660 |
| BRL-26 | Bryozoa | 18 16.54 S | 147 22.76 E | CWLTH | Island | 91 |  |  | 57503 |
| BRL-27 | Bryozoa | 18 16.54 S | 147 22.76 E | CWLTH | Island | 91 |  |  | 57530 |
| BRL-28 | Chlorophyta | 17 02.65 S | 119 37.87 E | CWLTH | Island | 271 |  |  | 51622 |
| BRL-29 | Chlorophyta | 17 02.65 S | 119 37.87 E | CWLTH | Island | 271 |  |  | 51623 |
| BRL-30 | Chlorophyta | 21 13.20 S | 152 00.20 E | QLD | Island | 164 |  |  | 54852 |
| BRL-31 | Chlorophyta | 18 16.27 S | 147 22.77 E | CWLTH | Island | 91 |  |  | 57508 |
| BRL-32 | Chlorophyta | 18 16.23 S | 147 22.85 E | CWLTH | Island | 91 |  |  | 57569 |
| BRL-33 | Chlorophyta | 18 16.23 S | 147 22.85 E | CWLTH | Island | 91 |  |  | 57570 |
| BRL-34 | Chlorophyta | 18 15.44 S | 147 23.01 E | CWLTH | Island | 93 |  |  | 57586 |
| BRL-35 | Chlorophyta | 18 16.27 S | 147 22.77 E | CWLTH | Island | 91 |  |  | 57594 |
| BRL-36 | Chlorophyta | 18 16.27 S | 147 22.77 E | CWLTH | Island | 91 |  |  | 57595 |
| BRL-37 | Chlorophyta | 18 16.23 S | 147 22.85 E | CWLTH | Island | 91 |  |  | 57599 |
| BRL-38 | Chlorophyta | 18 16.23 S | 147 22.85 E | CWLTH | Island | 91 |  |  | 57601 |
| BRL-39 | Chordata | 19 31.24 S | 117 26.04 E | CWLTH | Island | 107 |  |  | 51609 |
| BRL-40 | Chordata | 20 29.83 S | 117 20.98 E | CWLTH | Island | 20 |  |  | 51641 |
| BRL-41 | Chordata | 20 29.83 S | 117 20.98 E | CWLTH | Island | 20 |  |  | 51642 |
| BRL-42 | Chordata | 20 29.83 S | 117 20.98 E | CWLTH | Island | 20 |  |  | 51643 |
| BRL-43 | Chordata | 20 27.97 S | 117 17.77 E | CWLTH | Island | 16 |  |  | 51658 |
| BRL-44 | Chordata | 20 27.97 S | 117 17.77 E | CWLTH | Island | 16 |  |  | 51659 |
| BRL-45 | Chordata | 20 27.97 S | 117 17.77 E | CWLTH | Island | 16 |  |  | 51665 |
| BRL-46 | Chordata | 20 27.97 S | 117 17.77 E | CWLTH | Island | 16 |  |  | 51666 |
| BRL-47 | Chordata | 20 27.97 S | 117 17.77 E | CWLTH | Island | 16 |  |  | 51667 |
| BRL-48 | Chordata | 20 27.97 S | 117 17.77 E | CWLTH | Island | 16 |  |  | 51669 |
| BRL-49 | Chordata | 12 12.70 S | 122 59.99 E | CWLTH | Indonesia | 142 |  |  | 54835 |
| BRL-50 | Chordata | 21 33.72 S | 151 27.54 E | QLD | Island | 94 |  |  | 54844 |
| BRL-51 | Chordata | 21 33.72 S | 151 27.54 E | QLD | Island | 94 |  |  | 54875 |
| BRL-52 | Chordata | 12 14.07 S | 122 59.71 E | CWLTH | Indonesia | 144 |  |  | 54894 |
| BRL-53 | Chordata | 21 04.30 S | 151 36.20 E | CWLTH | Island | 138 |  |  | 54902 |
| BRL-54 | Chordata | 21 33.72 S | 151 27.54 E | QLD | Island | 94 |  |  | 54903 |
| BRL-55 | Chordata | 36 27.90 S | 150 18.48 E | CWLTH | Island | 24 |  |  | 57461 |
| BRL-56 | Chordata | 36 27.90 S | 150 18.48 E | CWLTH | Island | 24 |  |  | 57477 |
| BRL-57 | Chordata | 36 27.90 S | 150 18.48 E | CWLTH | Island | 24 |  |  | 57478 |
| BRL-58 | Chordata | 36 27.90 S | 150 18.48 E | CWLTH | Island | 24 |  |  | 57487 |
| BRL-59 | Chordata | 18 16.54 S | 147 22.76 E | CWLTH | Island | 91 |  |  | 57502 |
| BRL-60 | Chordata | 18 16.54 S | 147 22.76 E | CWLTH | Island | 91 |  |  | 57544 |
| BRL-61 | Chordata | 18 48.99 S | 147 37.97 E | CWLTH | Mainland | 60 |  |  | 57568 |
| BRL-62 | Chordata | 18 16.27 S | 147 22.77 E | CWLTH | Island | 91 |  |  | 57587 |
| BRL-63 | Chordata | 18 16.27 S | 147 22.77 E | CWLTH | Island | 91 |  |  | 57598 |
| BRL-64 | Cnidaria | 19 10.70 S | 117 42.18 E | CWLTH | Mainland | 138 |  |  | 51615 |
| BRL-65 | Cnidaria | 20 29.83 S | 117 20.98 E | CWLTH | Island | 20 |  |  | 51646 |
| BRL-66 | Cnidaria | 20 29.83 S | 117 20.98 E | CWLTH | Island | 20 |  |  | 51647 |
| BRL-67 | Cnidaria | 20 35.17 S | 117 16.26 E | CWLTH | Island | 8 |  |  | 51653 |
| BRL-68 | Cnidaria | 20 27.97 S | 117 17.77 E | CWLTH | Island | 16 |  |  | 51670 |
| BRL-69 | Cnidaria | 12 32.16 S | 123 34.75 E | CWLTH | Island | 173 |  |  | 54851 |
| BRL-70 | Cnidaria | 19 07.88 S | 146 52.47 E | QLD | Island | 0 | Beach | Full | 57468 |
| BRL-71 | Cnidaria | 19 07.88 S | 146 52.47 E | QLD | Island | 0 | Beach | Full | 57469 |
| BRL-72 | Cnidaria | 18 16.54 S | 147 22.76 E | CWLTH | Island | 91 |  |  | 57532 |
| BRL-73 | Cnidaria | 18 15.49 S | 147 22.80 E | CWLTH | Island | 92 |  |  | 57547 |
| BRL-74 | Cnidaria | 19 07.88 S | 146 52.47 E | QLD | Island | 0 | Beach | Full | 57560 |
| BRL-75 | Cnidaria | 19 07.88 S | 146 52.47 E | QLD | Island | 0 | Beach | Full | 57591 |
| BRL-76 | Cnidaria | 19 07.88 S | 146 52.47 E | QLD | Island | 0 | Beach | Full | 57593 |
| BRL-77 | Crustacea | 19 10.70 S | 117 42.18 E | CWLTH | Mainland | 138 |  |  | 51610 |
| BRL-78 | Crustacea | 19 10.70 S | 117 42.18 E | CWLTH | Mainland | 138 |  |  | 51611 |
| BRL-79 | Crustacea | 19 10.70 S | 117 42.18 E | CWLTH | Mainland | 138 |  |  | 51612 |
| BRL-80 | Crustacea | 19 10.70 S | 117 42.18 E | CWLTH | Mainland | 138 |  |  | 51613 |
| BRL-81 | Crustacea | 19 10.70 S | 117 42.18 E | CWLTH | Mainland | 138 |  |  | 51614 |
| BRL-82 | Echinodermata | 20 12.50 S | 116 55.62 E | CWLTH | Island | 19 |  |  | 51602 |
| BRL-83 | Echinodermata | 20 08.97 S | 116 59.41 E | CWLTH | Island | 28 |  |  | 51604 |
| BRL-84 | Echinodermata | 20 08.97 S | 116 59.41 E | CWLTH | Island | 28 |  |  | 51605 |
| BRL-85 | Echinodermata | 20 08.97 S | 116 59.41 E | CWLTH | Island | 28 |  |  | 51608 |
| BRL-86 | Echinodermata | 20 29.83 S | 117 20.98 E | CWLTH | Island | 20 |  |  | 51636 |
| BRL-87 | Miscellaneous | 19 22.54 S | 147 05.91 E | QLD | Mainland | 0 | Beach, Pandanus, Casuarina | Full | 57464 |
| BRL-88 | Miscellaneous | 19 22.54 S | 147 05.91 E | QLD | Mainland | 0 | Beach, Pandanus, Casuarina | Full | 57465 |
| BRL-89 | Miscellaneous | 19 22.54 S | 147 05.91 E | QLD | Mainland | 0 | Beach, Pandanus, Casuarina | Full | 57467 |
| BRL-90 | Miscellaneous | 19 22.54 S | 147 05.91 E | QLD | Mainland | 0 | Beach, Pandanus, Casuarina | Full | 57494 |
| BRL-91 | Miscellaneous | 19 22.54 S | 147 05.91 E | QLD | Mainland | 0 | Beach, Pandanus, Casuarina | Full | 57519 |
| BRL-92 | Miscellaneous | 19 22.54 S | 147 05.91 E | QLD | Mainland | 0 | Beach, Pandanus, Casuarina | Full | 57520 |
| BRL-93 | Miscellaneous | 19 22.54 S | 147 05.91 E | QLD | Mainland | 0 | Beach, Pandanus, Casuarina | Full | 57521 |
| BRL-94 | Miscellaneous | 19 22.54 S | 147 05.91 E | QLD | Mainland | 0 | Beach, Pandanus, Casuarina | Full | 57535 |
| BRL-95 | Miscellaneous | 19 22.54 S | 147 05.91 E | QLD | Mainland | 0 | Beach, Pandanus, Casuarina | Full | 57549 |
| BRL-96 | Miscellaneous | 19 22.54 S | 147 05.91 E | QLD | Mainland | 0 | Beach, Pandanus, Casuarina | Full | 57564 |
| BRL-97 | Mollusca | 21 04.30 S | 151 36.20 E | CWLTH | Island | 138 |  |  | 51652 |
| BRL-98 | Mollusca | 21 04.30 S | 151 36.20 E | CWLTH | Island | 138 |  |  | 51654 |
| BRL-99 | Mollusca | 21 29.41 S | 151 23.26 E | QLD | Island | 91 |  |  | 54850 |
| BRL-100 | Mollusca | 20 17.32 S | 115 22.95 E | CWLTH | Island | 17 |  |  | 57540 |
| BRL-101 | Phaeophyta | 17 04.92 S | 119 36.72 E | CWLTH | Island | 274 |  |  | 51628 |
| BRL-102 | Phaeophyta | 19 07.88 S | 146 52.47 E | QLD | Island | 0 | Beach | Full | 57558 |
| BRL-103 | Phaeophyta | 19 07.88 S | 146 52.47 E | QLD | Island | 0 | Beach | Full | 57559 |
| BRL-104 | Porifera | 20 12.50 S | 116 55.62 E | CWLTH | Island | 19 |  |  | 51598 |
| BRL-105 | Porifera | 20 12.50 S | 116 55.62 E | CWLTH | Island | 19 |  |  | 51599 |
| BRL-106 | Porifera | 20 12.50 S | 116 55.62 E | CWLTH | Island | 19 |  |  | 51600 |
| BRL-107 | Porifera | 20 12.50 S | 116 55.62 E | CWLTH | Island | 19 |  |  | 51601 |
| BRL-108 | Porifera | 20 12.50 S | 116 55.62 E | CWLTH | Island | 19 |  |  | 51603 |
| BRL-109 | Porifera | 20 08.97 S | 116 59.41 E | CWLTH | Island | 28 |  |  | 51606 |
| BRL-110 | Porifera | 17 04.87 S | 119 39.33 E | CWLTH | Island | 269 |  |  | 51618 |
| BRL-111 | Porifera | 17 04.87 S | 119 39.33 E | CWLTH | Island | 269 |  |  | 51620 |
| BRL-112 | Porifera | 17 04.92 S | 119 36.72 E | CWLTH | Island | 274 |  |  | 51625 |
| BRL-113 | Porifera | 17 04.92 S | 119 36.72 E | CWLTH | Island | 274 |  |  | 51626 |
| BRL-114 | Porifera | 17 04.92 S | 119 36.72 E | CWLTH | Island | 274 |  |  | 51627 |
| BRL-115 | Porifera | 17 09.73 S | 119 38.58 E | CWLTH | Island | 271 |  |  | 51629 |
| BRL-116 | Porifera | 17 09.73 S | 119 38.58 E | CWLTH | Island | 271 |  |  | 51630 |
| BRL-117 | Porifera | 17 09.73 S | 119 38.58 E | CWLTH | Island | 271 |  |  | 51631 |
| BRL-118 | Porifera | 17 09.73 S | 119 38.58 E | CWLTH | Island | 271 |  |  | 51632 |
| BRL-119 | Porifera | 17 09.73 S | 119 38.58 E | CWLTH | Island | 271 |  |  | 51633 |
| BRL-120 | Porifera | 20 29.83 S | 117 20.98 E | CWLTH | Island | 20 |  |  | 51634 |
| BRL-121 | Porifera | 20 29.83 S | 117 20.98 E | CWLTH | Island | 20 |  |  | 51635 |
| BRL-122 | Porifera | 20 29.83 S | 117 20.98 E | CWLTH | Island | 20 |  |  | 51638 |
| BRL-123 | Porifera | 20 29.83 S | 117 20.98 E | CWLTH | Island | 20 |  |  | 51639 |
| BRL-124 | Porifera | 20 29.83 S | 117 20.98 E | CWLTH | Island | 20 |  |  | 51640 |
| BRL-125 | Porifera | 20 29.83 S | 117 20.98 E | CWLTH | Island | 20 |  |  | 51644 |
| BRL-126 | Porifera | 20 29.83 S | 117 20.98 E | CWLTH | Island | 20 |  |  | 51645 |
| BRL-127 | Porifera | 20 29.53 S | 117 20.45 E | CWLTH | Island | 19 |  |  | 51650 |
| BRL-128 | Porifera | 20 35.17 S | 117 16.26 E | CWLTH | Island | 8 |  |  | 51651 |
| BRL-129 | Porifera | 20 29.53 S | 117 20.45 E | CWLTH | Island | 19 |  |  | 51655 |
| BRL-130 | Porifera | 20 29.53 S | 117 20.45 E | CWLTH | Island | 19 |  |  | 51657 |
| BRL-131 | Porifera | 20 27.97 S | 117 17.77 E | CWLTH | Island | 16 |  |  | 51662 |
| BRL-132 | Porifera | 20 27.97 S | 117 17.77 E | CWLTH | Island | 16 |  |  | 51663 |
| BRL-133 | Porifera | 20 27.97 S | 117 17.77 E | CWLTH | Island | 16 |  |  | 51664 |
| BRL-134 | Porifera | 20 27.97 S | 117 17.77 E | CWLTH | Island | 16 |  |  | 51673 |
| BRL-135 | Porifera | 20 27.97 S | 117 17.77 E | CWLTH | Island | 16 |  |  | 51674 |
| BRL-136 | Porifera | 20 01.22 S | 118 48.06 E | WA | Island | 1 |  |  | 51675 |
| BRL-137 | Porifera | 20 01.22 S | 118 48.06 E | WA | Island | 1 |  |  | 51676 |
| BRL-138 | Porifera | 20 01.22 S | 118 48.06 E | WA | Island | 1 |  |  | 51677 |
| BRL-139 | Porifera | 12 32.63 S | 123 34.14 E | CWLTH | Island | 172 |  |  | 54837 |
| BRL-140 | Porifera | 12 13.08 S | 123 00.22 E | CWLTH | Indonesia | 143 |  |  | 54842 |
| BRL-141 | Porifera | 12 32.63 S | 123 34.14 E | CWLTH | Island | 172 |  |  | 54843 |
| BRL-142 | Porifera | 14 11.68 S | 128 39.53 E | CWLTH | Island | 58 |  |  | 54876 |
| BRL-143 | Porifera | 14 11.68 S | 128 39.53 E | CWLTH | Island | 58 |  |  | 54895 |
| BRL-144 | Porifera | 14 11.68 S | 128 39.53 E | CWLTH | Island | 58 |  |  | 54897 |
| BRL-145 | Porifera | 18 50.57 S | 147 37.87 E | CWLTH | Mainland | 57 |  |  | 57471 |
| BRL-146 | Porifera | 18 33.64 S | 146 29.24 E | QLD | Island | 0 |  |  | 57476 |
| BRL-147 | Porifera | 18 16.27 S | 147 22.77 E | CWLTH | Island | 91 |  |  | 57480 |
| BRL-148 | Porifera | 18 15.49 S | 147 22.80 E | CWLTH | Island | 92 |  |  | 57500 |
| BRL-149 | Porifera | 18 16.54 S | 147 22.76 E | CWLTH | Island | 91 |  |  | 57504 |
| BRL-150 | Porifera | 18 16.27 S | 147 22.77 E | CWLTH | Island | 91 |  |  | 57507 |
| BRL-151 | Porifera | 18 15.49 S | 147 22.80 E | CWLTH | Island | 92 |  |  | 57526 |
| BRL-152 | Porifera | 18 50.06 S | 147 37.77 E | CWLTH | Mainland | 58 |  |  | 57528 |
| BRL-153 | Porifera | 18 16.54 S | 147 22.76 E | CWLTH | Island | 91 |  |  | 57541 |
| BRL-154 | Porifera | 18 16.27 S | 147 22.77 E | CWLTH | Island | 91 |  |  | 57542 |
| BRL-155 | Porifera | 18 16.54 S | 147 22.76 E | CWLTH | Island | 91 |  |  | 57543 |
| BRL-156 | Porifera | 18 15.49 S | 147 22.80 E | CWLTH | Island | 92 |  |  | 57545 |
| BRL-157 | Porifera | 18 15.49 S | 147 22.80 E | CWLTH | Island | 92 |  |  | 57546 |
| BRL-158 | Porifera | 18 16.54 S | 147 22.76 E | CWLTH | Island | 91 |  |  | 57551 |
| BRL-159 | Porifera | 18 15.49 S | 147 22.80 E | CWLTH | Island | 92 |  |  | 57556 |
| BRL-160 | Porifera | 18 16.54 S | 147 22.76 E | CWLTH | Island | 91 |  |  | 57561 |
| BRL-161 | Porifera | 18 15.49 S | 147 22.80 E | CWLTH | Island | 92 |  |  | 57571 |
| BRL-162 | Porifera | 18 16.27 S | 147 22.77 E | CWLTH | Island | 91 |  |  | 57575 |
| BRL-163 | Porifera | 18 15.49 S | 147 22.80 E | CWLTH | Island | 92 |  |  | 57596 |
| BRL-164 | Rhodophyta | 18 16.54 S | 147 22.76 E | CWLTH | Island | 91 |  |  | 57525 |
| BRL-165 | Rhodophyta | 18 16.54 S | 147 22.76 E | CWLTH | Island | 91 |  |  | 57529 |
| BRL-166 | Rhodophyta | 18 15.44 S | 147 23.01 E | CWLTH | Island | 93 |  |  | 57600 |
| BRL-167 | Sediment | 17 07.04 S | 146 20.35 E | CWLTH | Island | 29 |  |  | 51617 |
| BRL-168 | Sediment | 19 21.50 S | 147 02.00 E | QLD | Mainland | 0 | Intertidal | Full, Brine | 54853 |
| BRL-169 | Sediment | 19 21.50 S | 147 02.00 E | QLD | Mainland | 0 | Intertidal | Full, Brine | 54854 |
| BRL-170 | Sediment | 19 21.50 S | 147 02.00 E | QLD | Mainland | 0 | Intertidal | Full, Brine | 54855 |
| BRL-171 | Sediment | 19 21.50 S | 147 02.00 E | QLD | Mainland | 0 | Intertidal | Full, Brine | 54856 |
| BRL-172 | Sediment | 19 21.50 S | 147 02.00 E | QLD | Mainland | 0 | Intertidal | Full, Brine | 54857 |
| BRL-173 | Sediment | 19 21.50 S | 147 02.00 E | QLD | Mainland | 0 | Intertidal | Full, Brine | 54858 |
| BRL-174 | Sediment | 19 17.25 S | 147 01.60 E | QLD | Mainland | 0 | Tidal salt flats | Full | 54862 |
| BRL-175 | Sediment | 19 17.25 S | 147 01.60 E | QLD | Mainland | 0 | Tidal salt flats | Full | 54863 |
| BRL-176 | Sediment | 19 17.25 S | 147 01.60 E | QLD | Mainland | 0 | Tidal salt flats | Full | 54864 |
| BRL-177 | Sediment | 14 04.04 S | 121 37.73 E | CWLTH | Island | 211 |  |  | 54866 |
| BRL-178 | Sediment | 14 04.04 S | 121 37.73 E | CWLTH | Island | 211 |  |  | 54866 |
| BRL-179 | Sediment | 19 21.50 S | 147 02.00 E | QLD | Mainland | 0 | Intertidal | Full, Brine | 54873 |
| BRL-180 | Sediment | 19 21.50 S | 147 02.00 E | QLD | Mainland | 0 | Intertidal | Full, Brine | 54877 |
| BRL-181 | Sediment | 19 21.50 S | 147 02.00 E | QLD | Mainland | 0 | Intertidal | Full, Brine | 54878 |
| BRL-182 | Sediment | 19 21.50 S | 147 02.00 E | QLD | Mainland | 0 | Intertidal | Full, Brine | 54879 |
| BRL-183 | Sediment | 19 21.50 S | 147 02.00 E | QLD | Mainland | 0 | Intertidal | Full, Brine | 54881 |
| BRL-184 | Sediment | 19 21.50 S | 147 02.00 E | QLD | Mainland | 0 | Intertidal | Full, Brine | 54882 |
| BRL-185 | Sediment | 19 21.50 S | 147 02.00 E | QLD | Mainland | 0 | Intertidal | Full, Brine | 54884 |
| BRL-186 | Sediment | 19 21.50 S | 147 02.00 E | QLD | Mainland | 0 | Intertidal | Full, Brine | 54885 |
| BRL-187 | Sediment | 19 21.50 S | 147 02.00 E | QLD | Mainland | 0 | Intertidal | Full, Brine | 54886 |
| BRL-188 | Sediment | 19 21.50 S | 147 02.00 E | QLD | Mainland | 0 | Intertidal | Full, Brine | 54887 |
| BRL-189 | Sediment | 19 21.50 S | 147 02.00 E | QLD | Mainland | 0 | Intertidal | Full, Brine | 54888 |
| BRL-190 | Sediment | 19 21.50 S | 147 02.00 E | QLD | Mainland | 0 | Intertidal | Full, Brine | 54889 |
| BRL-191 | Sediment | 19 21.50 S | 147 02.00 E | QLD | Mainland | 0 | Intertidal | Full, Brine | 54890 |
| BRL-192 | Sediment | 19 21.50 S | 147 02.00 E | QLD | Mainland | 0 | Intertidal | Full, Brine | 54891 |
| BRL-193 | Sediment | 19 21.50 S | 147 02.00 E | QLD | Mainland | 0 | Intertidal | Full, Brine | 54892 |
| BRL-194 | Sediment | 19 21.50 S | 147 02.00 E | QLD | Mainland | 0 | Intertidal | Full, Brine | 54896 |
| BRL-195 | Sediment | 19 21.50 S | 147 02.00 E | QLD | Mainland | 0 | Intertidal | Full, Brine | 54898 |
| BRL-196 | Sediment | 19 21.50 S | 147 02.00 E | QLD | Mainland | 0 | Intertidal | Full, Brine | 54899 |
| BRL-197 | Sediment | 19 21.50 S | 147 02.00 E | QLD | Mainland | 0 | Intertidal | Full, Brine | 54900 |
| BRL-198 | Sediment | 15 45.99 S | 122 28.46 E | CWLTH | Island | 79 |  |  | 54901 |
| BRL-199 | Sediment | 19 06.00 S | 147 17.00 E | CWLTH | Mainland | 25 |  |  | 57460 |
| BRL-200 | Sediment | 19 22.00 S | 147 05.00 E | QLD | Mainland | 0 | Mangroves (establishing) | Full | 57470 |
| BRL-201 | Sediment | 19 06.00 S | 147 17.00 E | CWLTH | Mainland | 25 |  |  | 57472 |
| BRL-202 | Sediment | 19 12.00 S | 147 09.00 E | QLD | Island | 11 |  |  | 57473 |
| BRL-203 | Sediment | 19 00.00 S | 147 24.00 E | CWLTH | Mainland | 33 |  |  | 57474 |
| BRL-204 | Sediment | 19 14.98 S | 146 50.02 E | QLD | Mainland | 0 |  |  | 57475 |
| BRL-205 | Sediment | 19 14.98 S | 146 50.02 E | QLD | Mainland | 0 |  |  | 57479 |
| BRL-206 | Sediment | 19 22.00 S | 147 05.00 E | QLD | Mainland | 0 | Mangroves (establishing) | Full | 57484 |
| BRL-207 | Sediment | 19 22.00 S | 147 05.00 E | QLD | Mainland | 0 | Mangroves (establishing) | Full | 57486 |
| BRL-208 | Sediment | 19 06.00 S | 147 17.00 E | CWLTH | Mainland | 25 |  |  | 57488 |
| BRL-209 | Sediment | 19 22.54 S | 147 05.91 E | QLD | Mainland | 0 | Shallow shore, ebbing tide | Full | 57489 |
| BRL-210 | Sediment | 19 22.54 S | 147 05.91 E | QLD | Mainland | 0 | Shallow shore, ebbing tide | Full | 57490 |
| BRL-211 | Sediment | 19 22.00 S | 147 05.00 E | QLD | Mainland | 0 | Mangroves (establishing) | Full | 57491 |
| BRL-212 | Sediment | 19 22.00 S | 147 05.00 E | QLD | Mainland | 0 | Mangroves (establishing) | Full | 57492 |
| BRL-213 | Sediment | 19 22.54 S | 147 05.91 E | QLD | Mainland | 0 | Shallow shore, ebbing tide | Full | 57493 |
| BRL-214 | Sediment | 19 22.00 S | 147 05.00 E | QLD | Mainland | 0 | Mangroves (establishing) | Full | 57495 |
| BRL-215 | Sediment | 19 22.54 S | 147 05.91 E | QLD | Mainland | 0 | Shallow shore, ebbing tide | Full | 57496 |
| BRL-216 | Sediment | 19 00.00 S | 147 24.00 E | CWLTH | Mainland | 33 |  |  | 57497 |
| BRL-217 | Sediment | 19 12.00 S | 147 09.00 E | QLD | Island | 11 |  |  | 57498 |
| BRL-218 | Sediment | 18 52.00 S | 147 35.00 E | CWLTH | Mainland | 52 |  |  | 57499 |
| BRL-219 | Sediment | 19 22.00 S | 147 05.00 E | QLD | Mainland | 0 | Mangroves (establishing) | Full | 57501 |
| BRL-220 | Sediment | 18 59.90 S | 147 25.50 E | CWLTH | Mainland | 34 |  |  | 57505 |
| BRL-221 | Sediment | 19 06.10 S | 147 17.00 E | CWLTH | Mainland | 25 |  |  | 57506 |
| BRL-222 | Sediment | 19 22.54 S | 147 05.91 E | QLD | Mainland | 0 | Shallow shore, ebbing tide | Full | 57509 |
| BRL-223 | Sediment | 19 22.00 S | 147 05.00 E | QLD | Mainland | 0 | Mangroves (establishing) | Full | 57510 |
| BRL-224 | Sediment | 19 22.00 S | 147 05.00 E | QLD | Mainland | 0 | Mangroves (establishing) | Full | 57511 |
| BRL-225 | Sediment | 19 22.00 S | 147 05.00 E | QLD | Mainland | 0 | Mangroves (establishing) | Full | 57512 |
| BRL-226 | Sediment | 19 22.00 S | 147 05.00 E | QLD | Mainland | 0 | Mangroves (establishing) | Full | 57513 |
| BRL-227 | Sediment | 19 22.54 S | 147 05.91 E | QLD | Mainland | 0 | Shallow shore, ebbing tide | Full | 57514 |
| BRL-228 | Sediment | 18 51.95 S | 147 35.00 E | CWLTH | Mainland | 52 |  |  | 57522 |
| BRL-229 | Sediment | 19 06.10 S | 147 17.00 E | CWLTH | Mainland | 25 |  |  | 57527 |
| BRL-230 | Sediment | 18 51.95 S | 147 35.00 E | CWLTH | Mainland | 52 |  |  | 57531 |
| BRL-231 | Sediment | 19 22.00 S | 147 05.00 E | QLD | Mainland | 0 | Mangroves (establishing) | Full | 57533 |
| BRL-232 | Sediment | 19 12.00 S | 147 09.10 E | QLD | Island | 11 |  |  | 57536 |
| BRL-233 | Sediment | 19 22.00 S | 147 05.00 E | QLD | Mainland | 0 | Mangroves (establishing) | Full | 57537 |
| BRL-234 | Sediment | 19 22.00 S | 147 05.00 E | QLD | Mainland | 0 | Mangroves (establishing) | Full | 57538 |
| BRL-235 | Sediment | 19 22.54 S | 147 05.91 E | QLD | Mainland | 0 | Shallow shore, ebbing tide | Full | 57539 |
| BRL-236 | Sediment | 18 59.90 S | 147 25.50 E | CWLTH | Mainland | 34 |  |  | 57548 |
| BRL-237 | Sediment | 19 22.00 S | 147 05.00 E | QLD | Mainland | 0 | Mangroves (establishing) | Full | 57550 |
| BRL-238 | Sediment | 19 22.00 S | 147 05.00 E | QLD | Mainland | 0 | Mangroves (establishing) | Full | 57552 |
| BRL-239 | Sediment | 19 22.54 S | 147 05.91 E | QLD | Mainland | 0 | Shallow shore, ebbing tide | Full | 57553 |
| BRL-240 | Sediment | 19 22.54 S | 147 05.91 E | QLD | Mainland | 0 | Shallow shore, ebbing tide | Full | 57554 |
| BRL-241 | Sediment | 18 15.44 S | 147 23.01 E | CWLTH | Island | 93 |  |  | 57555 |
| BRL-242 | Sediment | 19 22.54 S | 147 05.91 E | QLD | Mainland | 0 | Shallow shore, ebbing tide | Full | 57562 |
| BRL-243 | Sediment | 19 22.54 S | 147 05.91 E | QLD | Mainland | 0 | Shallow shore, ebbing tide | Full | 57563 |
| BRL-244 | Sediment | 19 22.00 S | 147 05.00 E | QLD | Mainland | 0 | Mangroves (establishing) | Full | 57565 |
| 3-AIMS-BRL-LH | Crustacea | 13 50 21 | 146 39 20 | CWLTH | Island | 156 |  |  | 59787 |
| 5-AIMS-BRL-LH | Crustacea | 13 50 21 | 146 39 20 | CWLTH | Island | 156 |  |  | 59788 |
| 6-AIMS-BRL-LH | Crustacea | 13 55 27 | 146 42 47 | CWLTH | Island | 156 |  |  | 59789 |
| 9-AIMS-BRL-LH | Crustacea | 14 09 24 | 146 49 30 | CWLTH | Island | 157 |  |  | 59790 |
| 10-AIMS-BRL-LH | Crustacea | 14 09 24 | 146 49 30 | CWLTH | Island | 157 |  |  | 59791 |
| 16-AIMS-BRL-LH | Crustacea | 14 04 61 | 146 46 40 | CWLTH | Island | 155 |  |  | 59792 |
| 18-AIMS-BRL-LH | Crustacea | 13 55 27 | 146 42 47 | CWLTH | Island | 156 |  |  | 59793 |
| 19-AIMS-BRL-LH | Crustacea | 14 09 24 | 146 49 30 | CWLTH | Island | 157 |  |  | 59794 |
| 20-AIMS-BRL-LH | Crustacea | 14 09 24 | 146 49 30 | CWLTH | Island | 157 |  |  | 59795 |
| 22-AIMS-BRL-LH | Crustacea | 13 45 69 | 146 27 25 | CWLTH | Island | 144 |  |  | 59796 |
| 23-AIMS-BRL-LH | Chordata | 13 45 09 | 146 28 39 | CWLTH | Island | 147 |  |  | 59797 |
| 25-AIMS-BRL-LH | Chordata | 13 45 09 | 146 28 39 | CWLTH | Island | 147 |  |  | 59798 |
| 35-AIMS-BRL-LH | Chordata | 14 05 34 | 146 42 71 | CWLTH | Island | 148 |  |  | 59799 |
| 36-AIMS-BRL-LH | Chordata | 14 05 34 | 146 42 71 | CWLTH | Island | 148 |  |  | 59800 |
| 37-AIMS-BRL-LH | Chordata | 14 05 34 | 146 42 71 | CWLTH | Island | 148 |  |  | 59801 |
| 38-AIMS-BRL-LH | Chordata | 14 05 34 | 146 42 71 | CWLTH | Island | 148 |  |  | 59802 |
| 42-AIMS-BRL-LH | Crustacea | 14 09 24 | 146 49 30 | CWLTH | Island | 157 |  |  | 59803 |
| 45-AIMS-BRL-LH | Chordata | 14 05 34 | 146 42 71 | CWLTH | Island | 148 |  |  | 59804 |
| 46-AIMS-BRL-LH | Chordata | 14 05 34 | 146 42 71 | CWLTH | Island | 148 |  |  | 59805 |
| 50-AIMS-BRL-LH | Chordata | 14 06 87 | 146 44 31 | CWLTH | Island | 149 |  |  | 59806 |
| 51-AIMS-BRL-LH | Crustacea | 14 26 06 | 145 41 16 | CWLTH | Island | 32 |  |  | 59807 |
| 58-AIMS-BRL-LH | Crustacea | 14 26 06 | 145 41 16 | CWLTH | Island | 32 |  |  | 59808 |
| 60-AIMS-BRL-LH | Crustacea | 14 24 89 | 145 40 39 | CWLTH | Island | 32 |  |  | 59809 |
| 61-AIMS-BRL-LH | Crustacea | 14 24 89 | 145 40 39 | CWLTH | Island | 32 |  |  | 59810 |
| 63-AIMS-BRL-LH | Crustacea | 14 24 89 | 145 40 39 | CWLTH | Island | 32 |  |  | 59811 |
| 64-AIMS-BRL-LH | Crustacea | 14 26 06 | 145 41 16 | CWLTH | Island | 32 |  |  | 59812 |
| 66-AIMS-BRL-LH | Crustacea | 14 26 06 | 145 41 16 | CWLTH | Island | 32 |  |  | 59813 |
| 72-AIMS-BRL-LH | Crustacea | 13 48 79 | 144 40 54 | CWLTH | Island | 36 |  |  | 59814 |
| 84-AIMS-BRL-LH * | Crustacea | 14 24 89 | 145 40 39 | CWLTH | Island | 32 |  |  | 59815 |
| 85-AIMS-BRL-LH | Crustacea | 13 44 90 | 144 31 47 | CWLTH | Island | 39 |  |  | 59816 |
| 93-AIMS-BRL-LH * | Crustacea | 14 02 03 | 146 46 31 | CWLTH | Island | 157 |  |  | 59817 |
| 94-AIMS-BRL-LH * | Chordata | 14 02 37 | 146 40 04 | CWLTH | Island | 146 |  |  | 59818 |
| 98-AIMS-BRL-LH | Chordata | 13 42 15 | 144 14 73 | QLD | Island | 38 |  |  | 59819 |
| 99-AIMS-BRL-LH | Chordata | 14 02 37 | 146 40 04 | CWLTH | Island | 146 |  |  | 59820 |
| 102-AIMS-BRL-LH | Crustacea | 13 38 03 | 144 07 28 | QLD | Island | 44 |  |  | 59821 |
| 103-AIMS-BRL-LH | Chordata | 13 38 05 | 144 08 62 | QLD | Island | 46 |  |  | 59822 |
| 105-AIMS-BRL-LH | Crustacea | 14 02 02 | 146 44 11 | CWLTH | Island | 153 |  |  | 59823 |
| 106-AIMS-BRL-LH | Crustacea | 14 02 03 | 146 46 31 | CWLTH | Island | 157 |  |  | 59824 |
| 107-AIMS-BRL-LH | Chordata | 14 02 37 | 146 40 04 | CWLTH | Island | 146 |  |  | 59825 |
| 109-AIMS-BRL-LH | Crustacea | 14 00 47 | 146 45 40 | CWLTH | Island | 156 |  |  | 59826 |
| 110-AIMS-BRL-LH | Chaetognatha | 13 56 85 | 144 27 90 | QLD | Island | 18 |  |  | 59827 |
| 111-AIMS-BRL-LH | Crustacea | 14 02 03 | 146 46 31 | CWLTH | Island | 157 |  |  | 59828 |
| 112-AIMS-BRL-LH | Crustacea | 14 02 03 | 146 46 31 | CWLTH | Island | 157 |  |  | 59829 |
| 113-AIMS-BRL-LH | Chordata | 14 02 37 | 146 40 04 | CWLTH | Island | 146 |  |  | 59830 |
| 116-AIMS-BRL-LH | Crustacea | 14 00 47 | 146 45 40 | CWLTH | Island | 156 |  |  | 59831 |
| 117-AIMS-BRL-LH | Chaetognatha | 13 57 55 | 144 42 77 | QLD | Island | 27 |  |  | 59832 |
| 118-AIMS-BRL-LH | Crustacea | 14 00 47 | 146 45 40 | CWLTH | Island | 156 |  |  | 59833 |
| 119-AIMS-BRL-LH | Chordata | 13 42 15 | 144 14 73 | QLD | Island | 38 |  |  | 59834 |
| 120-AIMS-BRL-LH | Crustacea | 14 02 02 | 146 44 11 | CWLTH | Island | 153 |  |  | 59835 |
| 121-AIMS-BRL-LH | Crustacea | 14 02 02 | 146 44 11 | CWLTH | Island | 153 |  |  | 59836 |
| 122-AIMS-BRL-LH * | Crustacea | ** |  | CWLTH |  |  |  |  | 59837 |
| 123-AIMS-BRL-LH | Crustacea | ** |  | CWLTH |  |  |  |  | 59838 |
| 124-AIMS-BRL-LH | Chaetognatha | 13 57 55 | 144 42 77 | CWLTH | Island | 27 |  |  | 59839 |
| 125-AIMS-BRL-LH | Crustacea | ** |  | CWLTH |  |  |  |  | 59840 |
| 126-AIMS-BRL-LH | Crustacea | ** |  | CWLTH |  |  |  |  | 59841 |
| 127-AIMS-BRL-LH * | Crustacea | ** |  | CWLTH |  |  |  |  | 59842 |
| 128-AIMS-BRL-LH * | Crustacea | 14 00 47 | 146 45 40 | CWLTH | Island | 156 |  |  | 59843 |
| 129-AIMS-BRL-LH * | Chaetognatha | 13 57 55 | 144 42 77 | QLD | Island | 27 |  |  | 59844 |
| 130-AIMS-BRL-LH | Chordata | 13 38 05 | 144 08 62 | QLD | Island | 46 |  |  | 59845 |
| 131-AIMS-BRL-LH | Crustacea | ** |  | CWLTH |  |  |  |  | 59846 |
| 132-AIMS-BRL-LH | Chaetognatha | 13 57 55 | 144 42 77 | QLD | Island | 27 |  |  | 59847 |

Abbreviations: QLD: Queensland, Australia. CWLTH: Commonwealth of Australia

*Isolated using brain heart infusion agar prepared with mQ water instead of seawater

**Samples from aquaculture-reared lobster larvae fed natural prey items in on-board feeding experiment in the Coral Sea.
