# Supplementary material for: Diversity of Marine-Derived Fungal Cultures Exposed by DNA Barcodes: The Algorithm Matters
Source: PLoS One. 2015 Aug 26;10(8):e0136130. doi: 10.1371/journal.pone.0136130 (PMC4550264; doi:10.1371/journal.pone.0136130)
Supplement: S2 Table — Accession numbers of ITS barcode sequences, their best BLAST hit, and percentage sequence identity. (DOCX) [file pone.0136130.s011.docx]

Table S2: **BLAST results.** Accession numbers of ITS barcode sequences, their best BLAST hit, and percentage sequence identity.

| **Label** | **Source phylum** | **Accession number** | **Best BLAST hit (Accession number)** | **Sequence identity(%)** |
| --- | --- | --- | --- | --- |
| BRL-1 | Angiospermata | KP890479 | Aspergillus terreus (KC119206.1.1) | 100 |
| BRL-2 | Angiospermata | KP890537 | Setosphaeria rostrata (HE664033.1.1) | 100 |
| BRL-3 | Angiospermata | KP890538 | Setosphaeria rostrata (HE664033.1.1) | 100 |
| BRL-4 | Angiospermata | KP890643 | Trichoderma ghanense (EU280100.1) | 100 |
| BRL-5 | Angiospermata | KP890606 | Nigrospora sp. (JN207298.1) | 100 |
| BRL-6 | Angiospermata | KP890599 | Nigrospora sp. (JN207298.1) | 98.69 |
| BRL-7 | Angiospermata | KP890602 | Nigrospora sp. (JN207298.1) | 100 |
| BRL-8 | Angiospermata | KP890605 | Nigrospora sp. (JN207298.1) | 100 |
| BRL-9 | Angiospermata | KP890601 | Nigrospora sp. (JN207298.1) | 100 |
| BRL-10 | Angiospermata | KP890600 | Uncultured fungus clone (GQ999518.1) | 100 |
| BRL-11 | Angiospermata | KP890567 | Ampelomyces sp. (JQ36092.1) | 100 |
| BRL-12 | Angiospermata | KP890553 | Cochliobolus sp. CY126 (HQ607975.1) | 100 |
| BRL-13 | Angiospermata | KP890443 | Davidiella sp. (JX164075.1) | 100 |
| BRL-14 | Angiospermata | KP890535 | Uncultured Alternaria clone (JX159646.1) | 100 |
| BRL-15 | Angiospermata | KP890451 | Aspergillus niger (KC119204.1) | 100 |
| BRL-16 | Angiospermata | KP890539 | Setosphaeria rostrata (HE664033.1) | 100 |
| BRL-17 | Angiospermata | KP890442 | Aspergillus sp. (HE608807.1) | 100 |
| BRL-18 | Angiospermata | KP890429 | Emericella unguis (AB248983.1) | 99.65 |
| BRL-19 | Annelida | KP890502 | Uncultured Penicillium clone (KC143745.1) | 100 |
| BRL-20 | Annelida | KP890501 | Uncultured Penicillium clone (KC143745.1) | 100 |
| BRL-21 | Annelida | KP890505 | Uncultured Penicillium clone (KC143745.1) | 100 |
| BRL-22 | Bryozoa | KP890516 | Penicillium citrinum (JQ316514.1) | 100 |
| BRL-23 | Bryozoa | KP890503 | Uncultured Penicillium clone (KC143745.1) | 100 |
| BRL-24 | Bryozoa | KP890441 | Aspergillus sp. (HE608807.1) | 100 |
| BRL-25 | Bryozoa | KP890521 | Fungal sp. (JF330175.1) | 99.83 |
| BRL-26 | Bryozoa | KP890548 | Curvularia sp. Cv03Sc02 (JQ783059.1) | 99.83 |
| BRL-27 | Bryozoa | KP890564 | Cochliobolus sp. (JN207295.1) | 100 |
| BRL-30 | Chlorophyta | KP890635 | Fusarium sp. (JN207334.1) | 99.46 |
| BRL-31 | Chlorophyta | KP890589 | Cladosporium sp. (KC339216.1) | 100 |
| BRL-32 | Chlorophyta | KP890588 | Uncultured fungus clone (KC213757.1) | 100 |
| BRL-33 | Chlorophyta | KP890572 | Uncultured fungus clone (JX984771.1) | 99.82 |
| BRL-34 | Chlorophyta | KP890611 | Ascomycota sp. (JN561262.1) | 98.5 |
| BRL-35 | Chlorophyta | KP890504 | Uncultured Penicillium clone (KC143745.1) | 100 |
| BRL-36 | Chlorophyta | KP890644 | Trichoderma ghanense (EU280100.1) | 100 |
| BRL-37 | Chlorophyta | KP890604 | Nigrospora sp. (JN207298.1) | 100 |
| BRL-38 | Chlorophyta | KP890477 | Aspergillus terreus (KC119206.1) | 100 |
| BRL-39 | Chordata | KP890603 | Nigrospora sp. (JN207298.1) | 100 |
| BRL-40 | Chordata | KP890562 | Cochliobolus sp. P34E2 (JN207324.1) | 100 |
| BRL-42 | Chordata | KP890500 | Uncultured Penicillium clone (KC143745.1) | 100 |
| BRL-43 | Chordata | KP890520 | Fungal sp. (JF330175.1) | 99.83 |
| BRL-44 | Chordata | KP890590 | Uncultured fungus clone (GU721507.1) | 100 |
| BRL-45 | Chordata | KP890476 | Aspergillus terreus (KC119206.1) | 100 |
| BRL-46 | Chordata | KP890461 | Aspergillus niger (JX492319.1) | 99.83 |
| BRL-47 | Chordata | KP890484 | Penicillium verrucosum (KC009829.1) | 100 |
| BRL-48 | Chordata | KP890422 | Aspergillus variecolor (HQ674656.1) | 100 |
| BRL-49 | Chordata | KP890482 | Aspergillus sp. (HQ832962.1) | 97.53 |
| BRL-50 | Chordata | KP890478 | Aspergillus terreus (KC119206.1) | 100 |
| BRL-51 | Chordata | KP890629 | Fusarium sp. (HM535389.1) | 99.44 |
| BRL-52 | Chordata | KP890624 | Fusarium equiseti (HQ332532.1) | 99.81 |
| BRL-53 | Chordata | KP890532 | Alternaria sp. KJ-2012 (JN867467.1) | 100 |
| BRL-54 | Chordata | KP890623 | Fusarium sp. (JN207334.1) | 99.81 |
| BRL-55 | Chordata | KP890615 | Uncultured fungus clone (HQ257385.1) | 100 |
| BRL-56 | Chordata | KP890523 | Uncultured fungus clone (JX974742.1) | 99.42 |
| BRL-57 | Chordata | KP890612 | Acremonium implicatum (FN706541.1) | 99.82 |
| BRL-58 | Chordata | KP890513 | Penicillium citrinum (JQ316514.1) | 100 |
| BRL-59 | Chordata | KP890552 | Cochliobolus sp. CY126 (HQ607975.1) | 99.83 |
| BRL-60 | Chordata | KP890645 | Trichoderma longibrachiatum (KC009811.1) | 97.76 |
| BRL-61 | Chordata | KP890445 | Aspergillus sojae (AB008419.1) | 99.83 |
| BRL-62 | Chordata | KP890557 | Bipolaris sp. KH00278 (GU017499.1) | 100 |
| BRL-63 | Chordata | KP890583 | Leptosphaerulina chartarum (HQ607815.1) | 100 |
| BRL-64 | Cnidaria | KP890596 | Nigrospora sp. (GU017506.1) | 100 |
| BRL-65 | Cnidaria | KP890475 | Aspergillus terreus (KC119206.1) | 99.66 |
| BRL-66 | Cnidaria | KP890610 | Ascomycota sp. (JN561262.1) | 98.31 |
| BRL-67 | Cnidaria | KP890455 | Aspergillus tubingensis (JX501413.1) | 99.65 |
| BRL-68 | Cnidaria | KP890464 | Aspergillus niger (JX492319.1) | 99.66 |
| BRL-69 | Cnidaria | KP890628 | Fusarium sp. (HM535389.1) | 99.44 |
| BRL-70 | Cnidaria | KP890591 | Diaporthe phaseolorum (JQ936148.1) | 99.65 |
| BRL-71 | Cnidaria | KP890519 | Aspergillus fumigatus (HQ285578.1) | 100 |
| BRL-72 | Cnidaria | KP890481 | Aspergillus terreus (KC1192006.1) | 99.83 |
| BRL-73 | Cnidaria | KP890480 | Aspergillus terreus (KC119206.1) | 99.83 |
| BRL-74 | Cnidaria | KP890536 | Alternaria sp. MBP13A (JQ936168.1) | 100 |
| BRL-75 | Cnidaria | KP890571 | Cerebella andropogonis (AJ400905.1) | 99.62 |
| BRL-76 | Cnidaria | KP890463 | Aspergillus aff. (JN246057.1) | 99.83 |
| BRL-78 | Crustacea | KP890633 | Fusarium sp. (HM535389.1) | 99.44 |
| BRL-79 | Crustacea | KP890609 | Paecilomyces sp. (HQ631041.1) | 95.5 |
| BRL-80 | Crustacea | KP890474 | Aspergillus terreus (FR670307.1) | 99.66 |
| BRL-81 | Crustacea | KP890632 | Fusarium sp. (HM535389.1) | 99.44 |
| BRL-82 | Echinodermata | KP890531 | Alternaria sp. MUCL (AY714480.1) | 99.64 |
| BRL-83 | Echinodermata | KP890485 | Uncultured Penicillium clone (KC143745.1) | 98.94 |
| BRL-84 | Echinodermata | KP890518 | Aspergillus fumigatus (KC119200.1) | 99.49 |
| BRL-85 | Echinodermata | KP890636 | Fusarium sp. (GQ505438.1) | 98.74 |
| BRL-86 | Echinodermata | KP890619 | Fusarium solani (AF178407.1) | 97.28 |
| BRL-87 | Miscellaneous | KP890533 | Uncultured Alternaria clone (JX159646.1) | 100 |
| BRL-88 | Miscellaneous | KP890491 | Uncultured Penicillium clone (KC143745.1) | 99.83 |
| BRL-89 | Miscellaneous | KP890492 | Uncultured Penicillium clone (KC143745.1) | 99.83 |
| BRL-90 | Miscellaneous | KP890460 | Aspergillus niger (JX492319.1) | 99.83 |
| BRL-91 | Miscellaneous | KP890452 | Aspergillus niger (KC119204.1) | 99.83 |
| BRL-92 | Miscellaneous | KP890458 | Aspergillus tubingensis (JX501413.1) | 99.65 |
| BRL-93 | Miscellaneous | KP890466 | Aspergillus aff. (JN246057.1) | 99.65 |
| BRL-94 | Miscellaneous | KP890457 | Aspergillus tubingensis (JX501413.1) | 99.83 |
| BRL-95 | Miscellaneous | KP890425 | Aspergillus sp. (JN709036.1) | 99.82 |
| BRL-96 | Miscellaneous | KP890542 | Curvularia sp. Cv03Sc02 (JQ783059.1) | 99.83 |
| BRL-97 | Mollusca | KP890625 | Fusarium equiseti (HQ332532.1) | 99.81 |
| BRL-98 | Mollusca | KP890607 | Nigrospora sp. (JN207298.1) | 100 |
| BRL-99 | Mollusca | KP890556 | Bipolaris sp. KH00278 (GU017499.1) | 100 |
| BRL-100 | Mollusca | KP890555 | Cochliobolus sp. CY126 (HQ607975.1) | 99.83 |
| BRL-101 | Phaeophyta | KP890569 | Phoma sp. (GQ352490.1) | 98.84 |
| BRL-102 | Phaeophyta | KP890595 | Nigrospora sp. (HQ631070.1) | 99.45 |
| BRL-103 | Phaeophyta | KP890631 | Fusarium sp. (HM535389.1) | 99.44 |
| BRL-104 | Porifera | KP890622 | Fusarium sp. (JN207334.1) | 99.44 |
| BRL-105 | Porifera | KP890621 | Fusarium sp. (JN207334.1) | 99.25 |
| BRL-106 | Porifera | KP890540 | Setosphaeria rostrata (HE664033.1) | 99.83 |
| BRL-107 | Porifera | KP890554 | Cochliobolus sp. CY126 (HQ607975.1) | 100 |
| BRL-108 | Porifera | KP890573 | Phoma betae strain (EU594572.1) | 99.29 |
| BRL-109 | Porifera | KP890558 | Bipolaris sp. KH00278 (GU017499.1) | 100 |
| BRL-110 | Porifera | KP890630 | Fusarium sp. (HM535389.1) | 99.44 |
| BRL-111 | Porifera | KP890549 | Curvularia sp. Cv03Sc02 (JQ783059.1) | 99.65 |
| BRL-112 | Porifera | KP890565 | Uncultured fungus clone (GU721904.1) | 100 |
| BRL-113 | Porifera | KP890594 | Uncultured fungus clone (GQ999518.1) | 97.41 |
| BRL-114 | Porifera | KP890575 | Ascomycota sp. (FJ810163.1) | 99.29 |
| BRL-115 | Porifera | KP890620 | Fusarium sp. (JF740892.1) | 99.62 |
| BRL-116 | Porifera | KP890618 | Fusarium solani (AB775568.1) | 100 |
| BRL-117 | Porifera | KP890576 | Uncultured fungus clone (JX984754.1) | 99.82 |
| BRL-118 | Porifera | KP890462 | Aspergillus aff. (JN246057.1) | 99.83 |
| BRL-119 | Porifera | KP890459 | Aspergillus tubingensis (JX501413.1) | 99.65 |
| BRL-120 | Porifera | KP890608 | Nigrospora sp. (JN207298.1) | 100 |
| BRL-121 | Porifera | KP890568 | Ampelomyces sp. (JQ936092.1) | 99.61 |
| BRL-122 | Porifera | KP890592 | Pestalotiopsis microspora (HM012816.1) | 99.66 |
| BRL-123 | Porifera | KP890522 | Preussia sp. CY071 (HQ607945.1) | 98.85 |
| BRL-124 | Porifera | KP890488 | Uncultured Penicillium clone (KC143745.1) | 99.47 |
| BRL-125 | Porifera | KP890486 | Uncultured Penicillium clone (KC143745.1) | 99.12 |
| BRL-126 | Porifera | KP890487 | Uncultured Penicillium clone (KC143745.1) | 99.64 |
| BRL-127 | Porifera | KP890626 | Fusarium equiseti (HQ332532.1) | 99.81 |
| BRL-128 | Porifera | KP890634 | Fusarium sp. (HM535389.1) | 99.44 |
| BRL-130 | Porifera | KP890534 | Uncultured Alternaria clone (JX159646.1) | 100 |
| BRL-131 | Porifera | KP890638 | Tritirachium sp. (EU497949.1) | 99.83 |
| BRL-132 | Porifera | KP890616 | Emericellopsis terricola (FJ430737.1) | 98.76 |
| BRL-134 | Porifera | KP890515 | Penicillium citrinum (JQ316514.1) | 100 |
| BRL-135 | Porifera | KP890446 | Uncultured fungus clone (GU054043.1) | 99.82 |
| BRL-136 | Porifera | KP890494 | Uncultured Penicillium clone (KC143745.1) | 100 |
| BRL-137 | Porifera | KP890586 | Cladosporium cladosporioides (AB763555.1) | 98.52 |
| BRL-138 | Porifera | KP890584 | Cladosporium sp. (HE608784.1) | 99.82 |
| BRL-139 | Porifera | KP890508 | Penicillium citrinum (JQ316514.1) | 99.62 |
| BRL-140 | Porifera | KP890517 | Neosartorya fischeri (AB674772.2) | 98.96 |
| BRL-142 | Porifera | KP890456 | Aspergillus niger (JX492319.1) | 99.32 |
| BRL-143 | Porifera | KP890641 | Uncultured Trichoderma clone (JF449871.1) | 100 |
| BRL-144 | Porifera | KP890559 | Cochliobolus sp. P2E4 (JN207244.1) | 99.83 |
| BRL-145 | Porifera | KP890637 | Uncultured fungus clone (JF300442.1) | 99.27 |
| BRL-146 | Porifera | KP890507 | Penicillium citrinum (JQ316514.1) | 98.87 |
| BRL-147 | Porifera | KP890509 | Penicillium citrinum (JQ316514.1) | 99.82 |
| BRL-148 | Porifera | KP890639 | Tritirachium sp. (EU497949.1) | 99.83 |
| BRL-149 | Porifera | KP890444 | Aspergillus flavus (JX292092.1) | 100 |
| BRL-150 | Porifera | KP890470 | Aspergillus niger (JX492319.1) | 99.83 |
| BRL-151 | Porifera | KP890472 | Aspergillus niger (JX492319.1) | 100 |
| BRL-152 | Porifera | KP890454 | Aspergillus niger (KC119204.1) | 99.83 |
| BRL-153 | Porifera | KP890473 | Aspergillus niger ( JX492319.1) | 100 |
| BRL-154 | Porifera | KP890570 | Uncultured endophytic fungus (EF505848.1) | 98.87 |
| BRL-156 | Porifera | KP890587 | Uncultured fungus clone (KC213757.1) | 99.63 |
| BRL-157 | Porifera | KP890439 | Aspergillus sp. (JN709036.1) | 100 |
| BRL-158 | Porifera | KP890496 | Uncultured Penicillium clone (KC143745.1) | 100 |
| BRL-159 | Porifera | KP890420 | Aspergillus unguis (JN559768.1) | 99.82 |
| BRL-160 | Porifera | KP890430 | Syncephalastrum racemosum (HM999981.1) | 95.97 |
| BRL-161 | Porifera | KP890598 | Uncultured fungus clone (GQ999519.1) | 99.25 |
| BRL-162 | Porifera | KP890597 | Nigrospora sp. (GU017506.1) | 100 |
| BRL-163 | Porifera | KP890541 | Curvularia brachyspora (AF212308.1) | 96.64 |
| BRL-164 | Rhodophyta | KP890499 | Uncultured Penicillium clone (KC143745.1) | 100 |
| BRL-165 | Rhodophyta | KP890483 | Fungal sp. (JN709039.1) | 100 |
| BRL-166 | Rhodophyta | KP890471 | Aspergillus niger (JX492319.1) | 99.66 |
| BRL-167 | Sediment | KP890514 | Penicillium citrinum (JQ316514.1) | 100 |
| BRL-168 | Sediment | KP890431 | Syncephalastrum racemosum (HM999981.1) | 96.63 |
| BRL-169 | Sediment | KP890469 | Aspergillus niger (JX492319.1) | 99.83 |
| BRL-170 | Sediment | KP890440 | Aspergillus sp. (JN709036.1) | 100 |
| BRL-171 | Sediment | KP890527 | Uncultured fungus clone (EF596005.1) | 86.85 |
| BRL-172 | Sediment | KP890574 | Cf. Pleosporales sp. (HM596868.1) | 97.04 |
| BRL-173 | Sediment | KP890450 | Aspergillus niger (JX492319.1) | 99.15 |
| BRL-174 | Sediment | KP890419 | Aspergillus unguis (JN559768.1) | 99.82 |
| BRL-175 | Sediment | KP890418 | Aspergillus unguis (JN559768.1) | 100 |
| BRL-176 | Sediment | KP890525 | Uncultured fungus clone (EF596005.1) | 86.85 |
| BRL-177 | Sediment | KP890468 | Aspergillus niger (JX492319.1) | 99.83 |
| BRL-178 | Sediment | KP890560 | Cochliobolus sp. P2E4 (JN207244.1) | 99.32 |
| BRL-179 | Sediment | KP890582 | Paraphaeosphaeria sp. (HQ630993.1) | 97.2 |
| BRL-180 | Sediment | KP890551 | Curvularia sp. M5 (HM371207.1) | 97.92 |
| BRL-181 | Sediment | KP890489 | Uncultured Penicillium clone (KC143745.1) | 99.83 |
| BRL-182 | Sediment | KP890467 | Aspergillus niger (JX492319.1) | 99.83 |
| BRL-183 | Sediment | KP890528 | Uncultured fungus clone (EF596005.1) | 86.85 |
| BRL-184 | Sediment | KP890526 | Uncultured fungus clone ( EF596005.1) | 86.85 |
| BRL-185 | Sediment | KP890498 | Uncultured Penicillium clone (KC143745.1) | 100 |
| BRL-186 | Sediment | KP890435 | Aspergillus sp. (JN709036.1) | 100 |
| BRL-187 | Sediment | KP890613 | Acremonium sp. (JN207340.1) | 100 |
| BRL-188 | Sediment | KP890434 | Aspergillus sp. ( JN709036.1) | 100 |
| BRL-189 | Sediment | KP890426 | Aspergillus sp. (JN709036.1) | 99.82 |
| BRL-190 | Sediment | KP890436 | Aspergillus sp. ( JN709036.1) | 100 |
| BRL-191 | Sediment | KP890428 | Aspergillus caespitosus (KC253948.1) | 99.64 |
| BRL-192 | Sediment | KP890497 | Uncultured Penicillium clone (KC143745.1) | 100 |
| BRL-193 | Sediment | KP890490 | Penicillium chrysogenum (KC009826.1) | 100 |
| BRL-194 | Sediment | KP890438 | Aspergillus sp. (JN709036.1) | 100 |
| BRL-196 | Sediment | KP890614 | Acremonium sp. (JN207340.1) | 100 |
| BRL-198 | Sediment | KP890563 | Cochliobolus sp. (JN207325.1) | 99.82 |
| BRL-200 | Sediment | KP890524 | Uncultured fungus clone (EF596005.1) | 86.85 |
| BRL-201 | Sediment | KP890510 | Penicillium citrinum (JQ316514.1) | 99.82 |
| BRL-202 | Sediment | KP890493 | Uncultured Penicillium clone (KC143745.1) | 100 |
| BRL-203 | Sediment | KP890627 | Fusarium equiseti (JQ936153.1) | 100 |
| BRL-204 | Sediment | KP890529 | Phaeosphaeriopsis phacidiomorpha (FJ462742.1) | 86.76 |
| BRL-205 | Sediment | KP890547 | Curvularia sp. Cv03Sc02 (JQ783059.1) | 99.83 |
| BRL-206 | Sediment | KP890640 | Tritirachium sp. (EU497945.1) | 99.83 |
| BRL-207 | Sediment | KP890617 | Stachybotrys microspora (AF081475.2) | 99.13 |
| BRL-208 | Sediment | KP890581 | Coniothyrium sp. (JX624315.1) | 87.74 |
| BRL-209 | Sediment | KP890646 | Trichoderma longibrachiatum (KC009811.1) | 98.25 |
| BRL-210 | Sediment | KP890465 | Aspergillus niger (JX492319.1) | 99.66 |
| BRL-211 | Sediment | KP890437 | Aspergillus sp. (JN709036.1) | 100 |
| BRL-212 | Sediment | KP890447 | Penicillium sp. (JQ809682.1) | 97.88 |
| BRL-213 | Sediment | KP890512 | Penicillium citrinum (JQ316514.1) | 99.82 |
| BRL-214 | Sediment | KP890511 | Penicillium citrinum (JQ316514.1) | 99.82 |
| BRL-215 | Sediment | KP890433 | Aspergillus sp. (JN709036.1) | 100 |
| BRL-216 | Sediment | KP890566 | Cochliobolus lunatus (DQ836799.1) | 100 |
| BRL-217 | Sediment | KP890561 | Uncultured fungus clone (GU721911.1) | 98.39 |
| BRL-218 | Sediment | KP890546 | Curvularia sp. Cv03Sc02 (JQ783059.1) | 99.83 |
| BRL-219 | Sediment | KP890543 | Curvularia sp. Cv03Sc02 (JQ783059.1) | 99.83 |
| BRL-220 | Sediment | KP890506 | Penicillium citrinum (JQ316514.1) | 98.71 |
| BRL-222 | Sediment | KP890550 | Curvularia sp. Cv03Sc02 (JQ783059.1) | 99.65 |
| BRL-223 | Sediment | KP890579 | Uncultured fungus clone (GQ999275.1) | 99.46 |
| BRL-224 | Sediment | KP890593 | Arthrinium sp. (AB462755.1) | 97.04 |
| BRL-225 | Sediment | KP890423 | Emericella sp. (JN689343.1) | 100 |
| BRL-227 | Sediment | KP890578 | Periconia sp. (DQ336713.1) | 99.44 |
| BRL-228 | Sediment | KP890495 | Uncultured Penicillium clone (KC143745.1) | 100 |
| BRL-229 | Sediment | KP890453 | Aspergillus niger (KC119204.1) | 99.83 |
| BRL-230 | Sediment | KP890449 | Aspergillus niger (JX492319.1) | 98 |
| BRL-231 | Sediment | KP890448 | Aspergillus sp. (HM801881.1) | 98.13 |
| BRL-232 | Sediment | KP890424 | Aspergillus sydowii (KC253961.1) | 100 |
| BRL-233 | Sediment | KP890580 | Uncultured fungus clone (GQ999275.1) | 99.64 |
| BRL-234 | Sediment | KP890585 | Cladosporium sp. (HE608784.1) | 100 |
| BRL-235 | Sediment | KP890544 | Curvularia sp. Cv03Sc02 (JQ783059.1) | 99.83 |
| BRL-236 | Sediment | KP890530 | Alternaria sp. KJ-2012 (JN867467.1) | 99.64 |
| BRL-237 | Sediment | KP890545 | Curvularia sp. Cv03Sc02 (JQ783059.1) | 99.83 |
| BRL-239 | Sediment | KP890421 | Aspergillus unguis (JN559768.1) | 99.82 |
| BRL-240 | Sediment | KP890427 | Penicillium minioluteum (FR670332.1) | 97.93 |
| BRL-241 | Sediment | KP890642 | Hypocrea lixii (JN108918.1) | 100 |
| BRL-243 | Sediment | KP890577 | Ascomycota sp. (HQ608101.1) | 100 |
| BRL-244 | Sediment | KP890432 | Aspergillus versicolor (FJ878625.1) | 100 |
| 3-AIMS-BRL-LH | Crustacea | KP890375 | Uncultured fungus clone (KC213757.1) | 100 |
| 5-AIMS-BRL-LH | Crustacea | KP890378 | Gibberella moniliformis (HQ316575.1) | 100 |
| 6-AIMS-BRL-LH | Crustacea | KP890417 | Emericella unguis (AB428983.1) | 99.63 |
| 9-AIMS-BRL-LH | Crustacea | KP890362 | Didymellaceae sp. (JQ717314.1) | 99.82 |
| 10-AIMS-BRL-LH | Crustacea | KP890408 | Penicillium verrucosum (KC009829.1) | 99.82 |
| 16-AIMS-BRL-LH | Crustacea | KP890390 | Fusarium dimerum (JQ434586.1.1) | 100 |
| 18-AIMS-BRL-LH | Crustacea | KP890359 | Tilletiopsis minor (AB025702.1) | 92.76 |
| 19-AIMS-BRL-LH | Crustacea | KP890377 | Gibberella moniliformis (HQ316575.1) | 99.8 |
| 20-AIMS-BRL-LH | Crustacea | KP890379 | Gibberella moniliformis (HQ316575.1) | 100 |
| 22-AIMS-BRL-LH | Crustacea | KP890385 | Aspergillus penicillioides (GU017496.1) | 99.66 |
| 23-AIMS-BRL-LH | Chordata | KP890383 | Candida sp. (EU818716.1) | 87.87 |
| 25-AIMS-BRL-LH | Chordata | KP890402 | Penicillium verrucosum (KC009829.1) | 100 |
| 35-AIMS-BRL-LH | Chordata | KP890361 | Tritirachium oryzae (JF779661.1) | 100 |
| 36-AIMS-BRL-LH | Chordata | KP890391 | Microascus sp. (AB566304.1) | 100 |
| 37-AIMS-BRL-LH | Chordata | KP890368 | Hortaea sp. (FJ755827.1) | 100 |
| 38-AIMS-BRL-LH | Chordata | KP890380 | Gibberella moniliformis (HQ316575.1) | 100 |
| 42-AIMS-BRL-LH | Crustacea | KP890360 | Tilletiopsis minor (AB025702.1) | 92.76 |
| 45-AIMS-BRL-LH | Chordata | KP890374 | Fungal endophyte (EU687048.1) | 84.97 |
| 46-AIMS-BRL-LH | Chordata | KP890381 | Gibberella moniliformis (HQ316575.1) | 100 |
| 50-AIMS-BRL-LH | Chordata | KP890372 | Hortaea sp. (FJ755827.1) | 100 |
| 51-AIMS-BRL-LH | Crustacea | KP890386 | Colletotrichum tropicale (JX010275.1) | 99.81 |
| 58-AIMS-BRL-LH | Crustacea | KP890388 | Tritirachium sp. (JQ717341.1) | 100 |
| 60-AIMS-BRL-LH | Crustacea | KP890387 | Colletotrichum gloeosporioides (KC010541.1) | 99.78 |
| 61-AIMS-BRL-LH | Crustacea | KP890363 | Guignardia mangiferae (JQ743587.1) | 99.35 |
| 63-AIMS-BRL-LH | Crustacea | KP890371 | Hortaea sp. (FJ755827.1) | 99.2 |
| 64-AIMS-BRL-LH | Crustacea | KP890373 | Gibberella moniliformis (HQ316575.1) | 99.6 |
| 66-AIMS-BRL-LH | Crustacea | KP890414 | Aschersonia tamurai (DQ347965.1) | 100 |
| 72-AIMS-BRL-LH | Crustacea | KP890384 | Candida parapsilosis (GQ395610.1) | 100 |
| 84-AIMS-BRL-LH | Crustacea | KP890364 | Dothideomycetes sp. (JQ760395.1) | 100 |
| 85-AIMS-BRL-LH | Crustacea | KP890415 | Aschersonia tamurai (DQ347965.1) | 100 |
| 93-AIMS-BRL-LH | Crustacea | KP890382 | Uncultured fungus (JX174836.1) | 100 |
| 94-AIMS-BRL-LH | Chordata | KP890399 | Penicillium paxilli (JN617709.1) | 99.45 |
| 98-AIMS-BRL-LH | Chordata | KP890366 | Teratosphaeria sp. (JN709043.1) | 98.64 |
| 99-AIMS-BRL-LH | Chordata | KP890370 | Cladosporium colombiae (KC339772.1) | 100 |
| 102-AIMS-BRL-LH | Crustacea | KP890389 | Acremonium charticola (AJ621774.1) | 93.1 |
| 103-AIMS-BRL-LH | Chordata | KP890365 | Ramichloridium brasilianum (EU041797.1) | 99.03 |
| 105-AIMS-BRL-LH | Crustacea | KP890403 | Penicillium sp. (HQ738282.1) | 99.45 |
| 106-AIMS-BRL-LH | Crustacea | KP890376 | Cladosporium sp. (HE608784.1) | 100 |
| 107-AIMS-BRL-LH | Chordata | KP890412 | Candida sp. (FJ008051.1) | 99.82 |
| 109-AIMS-BRL-LH | Crustacea | KP890411 | Candida parapsilosis (GQ395610.1) | 100 |
| 110-AIMS-BRL-LH | Chaetognatha | KP890398 | Aspergillus niger (JQ764805.1) | 99.82 |
| 111-AIMS-BRL-LH | Crustacea | KP890367 | Hortaea sp. (FJ755827.1) | 100 |
| 112-AIMS-BRL-LH | Crustacea | KP890413 | Debaryomyces hansenii (JQ912667.1) | 100 |
| 113-AIMS-BRL-LH | Chordata | KP890406 | Penicillium sp. (HQ738282.1) | 99.63 |
| 116-AIMS-BRL-LH | Crustacea | KP890358 | Tilletiopsis minor (AB025702.1) | 92.4 |
| 117-AIMS-BRL-LH | Chaetognatha | KP890369 | Cladosporium perangustum (JQ863247.1) | 100 |
| 118-AIMS-BRL-LH | Crustacea | KP890357 | Tilletiopsis minor (AB025702.1) | 92.64 |
| 119-AIMS-BRL-LH | Chordata | KP890392 | Aspergillus sp. (AM901671.1) | 99.82 |
| 120-AIMS-BRL-LH | Crustacea | KP890416 | Debaryomyces hansenii (JQ912667.1) | 99.83 |
| 121-AIMS-BRL-LH | Crustacea | KP890405 | Penicillium sp. (HQ738282.1) | 99.45 |
| 122-AIMS-BRL-LH | Crustacea | KP890404 | Penicillium chrysogenum (JF440603.1) | 100 |
| 123-AIMS-BRL-LH | Crustacea | KP890393 | Penicillium chrysogenum (JF440603.1) | 100 |
| 124-AIMS-BRL-LH | Chaetognatha | KP890394 | Penicillium chrysogenum (JF440603.1) | 100 |
| 125-AIMS-BRL-LH | Crustacea | KP890410 | Aspergillus caelatus (JQ676205.1) | 95.74 |
| 126-AIMS-BRL-LH | Crustacea | KP890407 | Penicillium chrysogenum (JF440603.1) | 96.91 |
| 127-AIMS-BRL-LH | Crustacea | KP890395 | Penicillium chrysogenum (JF440603.1) | 100 |
| 128-AIMS-BRL-LH | Crustacea | KP890396 | Penicillium chrysogenum (JF440603.1) | 100 |
| 129-AIMS-BRL-LH | Chaetognatha | KP890400 | Penicillium sp. (HQ738282.1) | 99.63 |
| 130-AIMS-BRL-LH | Chordata | KP890409 | Uncultured fungus clone (JF497127.1) | 99.47 |
| 131-AIMS-BRL-LH | Crustacea | KP890397 | Penicillium chrysogenum (JF440603.1) | 100 |
| 132-AIMS-BRL-LH | Chaetognatha | KP890401 | Penicillium sp. (HQ738282.1) | 99.63 |
